# Intrafloral patterns of color and scent in *Capparis spinosa* L. and the ghosts of its selection past

**DOI:** 10.1002/ajb2.16098

**Published:** 2022-12-15

**Authors:** Aphrodite Kantsa, Jair E. Garcia, Robert A. Raguso, Adrian G. Dyer, Ronny Steen, Thomas Tscheulin, Theodora Petanidou

**Affiliations:** ^1^ Department of Geography University of the Aegean Mytilene Greece; ^2^ Bio‐Inspired Digital Sensing Laboratory, School of Media and Communication RMIT University Melbourne Australia; ^3^ Department of Neurobiology and Behavior Cornell University, Ithaca New York USA; ^4^ Department of Physiology Monash University Clayton Australia; ^5^ Department of Ecology and Natural Resource Management Norwegian University of Life Sciences Ås Norway; ^6^ Present address: Department of Environmental Systems Science ETH Zürich Zürich Switzerland; ^7^ Present address: Department of Developmental Biology and Neurobiology Johannes Gutenberg University Mainz Germany

**Keywords:** aldoximes, brush flowers, Capparaceae, carpenter bees, glucosinolates, hawkmoths, mixed pollination systems, nectar guides, nocturnal pollination, pollinator vision

## Abstract

**Premise:**

*Capparis spinosa* is a widespread charismatic plant, in which the nocturnal floral habit contrasts with the high visitation by diurnal bees and the pronounced scarcity of hawkmoths. To resolve this discrepancy and elucidate floral evolution of *C. spinosa*, we analyzed the intrafloral patterns of visual and olfactory cues in relation to the known sensory biases of the different visitor guilds (bees, butterflies, and hawkmoths).

**Methods:**

We measured the intrafloral variation of scent, reflectance spectra, and colorimetric properties according to three guilds of known visitors of *C. spinosa*. Additionally, we sampled visitation rates using a motion‐activated camera.

**Results:**

Carpenter bees visited the flowers eight times more frequently than nocturnal hawkmoths, at dusk and in the following morning. Yet, the floral headspace of *C. spinosa* contained a typical sphingophilous scent with high emission rates of certain monoterpenes and amino‐acid derived compounds. Visual cues included a special case of multisensory nectar guide and color patterns conspicuous to the visual systems of both hawkmoths and bees.

**Conclusions:**

The intrafloral patterns of sensory stimuli suggest that hawkmoths have exerted strong historical selection on *C. spinosa*. Our study revealed two interesting paradoxes: (a) the flowers phenotypically biased towards the more inconsistent pollinator; and (b) floral display demands an abundance of resources that seems maladaptive in the habitats of *C. spinosa*. The transition to a binary pollination system accommodating large bees has not required phenotypic changes, owing to specific eco‐physiological adaptations, unrelated to pollination, which make this plant an unusual case in pollination ecology.

Pollinators are agents of selection on floral phenotypes both at microevolutionary and macroevolutionary levels (van der Niet and Johnson, 2012; van der Niet et al., 2014; Phillips et al., 2020). In this context, specific combinations of floral traits are known to predict extant pollinator niches reflecting diffuse co‐evolutionary affiliations. In some cases, predictions are particularly straightforward and accurate, as for example in hawkmoth‐pollinated or “sphingophilous” plants (Knudsen and Tollsten, 1993; Whittall and Hodges, 2007; Johnson and Raguso, 2016; Johnson and Wester, 2017), or in the oil‐secreting Coryciinae orchids (Pauw, 2006). However, the question of whether floral phenotypes may in general predict the principal pollinator is a long‐standing debate (Wallace, 1867; Müller and Delpino, 1871; Vogel, 1954; Stebbins, 1970; Fægri and Van der Pijl, 1979; Waser et al., 1996; Aigner, 2001; Fenster et al., 2004; Ollerton et al., 2009; Rosas‐Guerrero et al., 2014). Indeed, the adherence to strict predictions or interpretations of the “syndrome” concept runs the risk of overlooking important aspects of floral reproductive ecology and evolution, e.g., the roles of phylogenetic constraints, abiotic factors, alternative agents of selection, mixed mating systems, the potential for binary or generalized pollination systems, reproductive assurance, or the impact of opportunistic species (Waser et al., 1996; Sun et al., 2008).

Examples of seemingly opportunistic flowers are those accessible to multiple pollinator groups owing to their architecture and phenology. In this context, an interesting group of “open” flowers, in which rewards are easily accessible to a wide range of animals, are the brush‐flowers, i.e., flowers (or inflorescences) with numerous protruding long stamens and styles, lacking a prominent nectar tube (Werth, 1942). Although clearly defined, brush‐flowers represent a peculiar case in the natural history of pollination. At first, they were considered as primarily adapted to visitors with long mouth parts that carry pollen mainly on their abdomen, e.g., hummingbirds or hawkmoths (Fægri and Van der Pijl, 1979). The spatial separation of sexual organs and nectar is the reason why brush‐flowers can be sphingophilous (even though the corolla is not funnel‐shaped), resulting in pollen placement on wings or bodies instead of strictly on the proboscis or head (Baum, 1995; Moré et al., 2006). Yet, among sphingophilous taxa, brush‐flowers are functional outliers precisely because the copious nectar is easily reachable even by short‐tongued visitors (Johnson et al., 2017). As a consequence, brush‐flowers are usually exploited by a wide range of animals (Schneck, 1895; Haber and Frankie, 1982; Koptur, 1983; Eisikowitch et al., 1986; Petanidou, 1991; Gribel and Hay, 1993; Silva and Sazima, 1995; Groman and Pellmyr, 1999; Locatelli and Machado, 1999; Quesada et al., 2001; Oliveira et al., 2004; Machado et al., 2006; Moré et al., 2006; Zhang and Tan, 2009; Amorim et al., 2013), representing textbook examples of flowers that appear to lack specialized evolutionary connections with pollinator guilds (Kers, 2003; Willmer, 2011).

The family Capparaceae includes typical brush‐flowered species that are thought to have evolved from Papaveraceae‐like choripetalous ancestors (Fægri and Van der Pijl, 1979). *Capparis spinosa* L. (caper bush) is the most prominent member of the family, a plant with high phytochemical potential, economic value, and ethnobotanical significance dating to the Neolithic Period (Jiang et al., 2007; Chedraoui et al., 2017). Despite its importance, there are open questions regarding its markedly unclear biogeographical status, and its reproductive ecology.

Ecological research has indicated a mixed pollination system including predominantly bees and, less often, nocturnal hawkmoths (Dafni et al., 1987; Petanidou, 1991). However, noting the absence of known flower‐visiting bats in the Palearctic realm (Fleming et al., 2009), the floral phenotype of *C. spinosa* (Appendix S1, Figure S1) indicates a rather straightforward prediction for hawkmoth pollination, given the (1) brush‐type flowers, (2) strictly nocturnal anthesis, strong scent emissions, and abundant nectar secretion, as well as (3) the hawkmoth‐biased sucrose:hexose ratio of nectar (Baker and Baker, 1990; Petanidou et al., 1996). Nonetheless, if not for the nocturnal anthesis, it might be easy to overlook the potential significance of hawkmoths for *C. spinosa*, owing to their highly irregular presence as observed floral visitors (Eisikowitch et al., 1986; Dafni et al., 1987; Petanidou, 1991; Petanidou et al., 1996; Zhang and Tan, 2009). As a result, even though hawkmoths are at least as efficient as bees in pollinating *C. spinosa* (Dafni et al., 1987), their importance has been undervalued. For instance, the copious nocturnal secretion of nectar was previously attributed to intraspecific competition between the (short‐lived) flowers for diurnal pollinators (Petanidou et al., 1996).

Given the apparent discrepancy between floral display and pollinator visitation, we aimed to examine floral color and scent of *C. spinosa*, and test whether these are associated with known sensory biases of the different visitor guilds. Because flowers represent complex, multimodal phenotypes and may contain nested signals, such as color contrast or scented rewards (Leonard et al., 2012), we analyzed the intrafloral variation in visual and olfactory aspects of floral display. Specifically, we measured (a) reflectance spectra and color according to the available models of visual perception for four different classes of pollinating animals, and (b) the constitutive emissions of volatile compounds from different flower parts. We hypothesized that the sensory cues associated with floral morphology, anthesis time, and nectar production should match the known preferences of nocturnal hawkmoths. For instance, we expected a high representation of acyclic terpenoids, nitrogenous and benzenoid volatile organic compounds (VOCs), which are known to be emitted by hawkmoth‐pollinated flowers worldwide (Knudsen and Tollsten, 1993; Levin et al., 2003; Steen et al., 2019). Moreover, we hypothesized the presence of hawkmoth‐specific, multimodal nectar guides according to hawkmoth sensory requirements, which are known to optimize correct proboscis placement, alignment of the body and proboscis with the nectary, and probing duration (e.g., Goyret and Raguso, 2006; Goyret, 2010; Goyret and Yuan, 2015). Additionally, we video‐monitored floral visitors on Lesvos Island, Greece, to quantify visitation in an unbiased way. Overall, this approach is based on the need to include sensory floral traits in studying plant–pollinator coevolution (Dyer et al., 2012), and it can provide evidence to elucidate the selective agents for *C. spinosa*.

## MATERIALS AND METHODS

### Study species

*Capparis spinosa* L. is an andromonoecious perennial plant with large, white, zygomorphic, brush‐type flowers that last for one night (Appendix 1, Figure S1). It is often highlighted that several of its floral traits are plesiomorphic (e.g., solitary axillary flowers, large ovary with numerous carpels, centrifugally arranged multi‐staminate androecia), implying an ancient (sub‐)tropical origin and primitive status among the Capparaceae (Ronse De Craene and Smets, 1997; Fici, 2004; Naghiloo et al., 2015).

Despite its charisma and prominent role in phytochemical research (Chedraoui et al., 2017), our basic knowledge of *C. spinosa* is fragmentary, starting with its taxonomy. Although *C. spinosa* represents the type species of Capparaceae, it has been characterized as a “blanket identification” of numerous undefined taxonomic entities within the genus, or as a nothospecies, i.e., a hybrid kept in cultivation in and around the Mediterranean Basin (Zohary, 1973; Inocencio et al., 2006). As a result, the “chaotic” taxonomic status of *C. spinosa* covers the entire lumper–splitter continuum (Zohary, 1960; Jacobs, 1965; Fici, 2001; Inocencio et al., 2006; Fici, 2014).

Equally intriguing is that the biogeographical origin of *C. spinosa* remains a mystery. Based on its extant distribution, *C. spinosa* is considered a pan‐ and sub‐tropical taxon, spanning from the Mediterranean Basin southwards to sub‐Saharan Africa, and across Asia to Oceania (Jacobs, 1965). Domestication has generally been excluded (see Fici, 2004), although we do not fully embrace this view. The most popular hypotheses suggest that it is a relic of a xero‐tropical flora from the Tertiary (Paleocene to Pliocene), probably originating in Africa or western Asia (Zohary, 1973; Fici, 2001).

### Study sites

The present study took place on Lesvos Island, NE Greece. Specifically, we studied three wild populations of *C. spinosa*, in Thermi (scent and color sampling; 39° 10′54″N, 26° 29′52″E), in Koudouroudia (scent sampling; 39°02′46″N, 26°30′58″E), and in Mytilene (visitor recording; 39°05′09″N, 26°34′08″E); the minimum distance between sites was 8.6 km, and the maximal was 20.7 km. Along this distance, the plants’ habit, landscape, elevation, climate (thermo‐Mediterranean), natural vegetation (Mediterranean‐type scrublands including phrygana and low maquis), as well as the pollinator fauna (Dauber et al., 2010; Nielsen et al., 2011; “The Melissotheque of the Aegean”, Petanidou et al., unpublished) are invariant. Thus, our sampling sites represent one primary population of *C. spinosa* of the eastern part of Lesvos Island.

### Floral reflectance and colorimetry

#### Reflectance spectra

The flower of *C. spinosa* has three distinctly colored regions, which we sampled for reflectance spectra: (a) the petals (white for humans); (b) the distal half of the filaments of the stamens (pink‐magenta); and (c) the green basal parts of the dorsal pair of petals providing access to the nectary (Appendix S1, Figure S2). We measured the reflectance spectra (300–700 nm) from seven healthy, fresh flowers of different individuals in the population at Thermi, using a portable Jaz spectrometer equipped with a Premium 600 μm reflectance probe (Ocean Optics, Orlando, Florida, USA).

At the spectral level, we calculated mean brightness, which is considered important for nocturnal hawkmoths (van der Kooi and Kelber, 2022), expressing the mean relative reflectance over the entire spectral range. For this, we used the function *summary()* of the R package pavo 2.4.0 (Maia et al., 2019). To estimate the variation of brightness among the three floral parts, we applied a Friedman test for repeated measures using the function *friedman_test()* of the R package rstatix 0.7.0 (Kassambara, 2022), followed by post‐hoc pairwise Wilcoxon signed‐rank tests with Bonferroni correction.

#### Colorimetric analysis

Color is a perception defined according to a specific visual system. Thus, since different pollinators may perceive the reflected light differently, we measured variation in the colorimetric properties of the floral parts of *C. spinosa*, relevant to the visual systems of its known floral visitors. Using the acquired reflectance spectra of the floral parts, we modeled photoreceptor stimulation according to four visual systems that correspond to different pollinator guilds, specifically:

1. *Nocturnal hawkmoths (predicted pollinator, low frequency)*. We employed the visual system of the tobacco hornworm hawkmoth (*Manduca sexta* L. [Sphingidae]), which has been extensively studied. Tobacco hornworms have three types of photoreceptors in the ventral part of their retina, which is considered as functionally specialized for foraging: UV (maximal sensitivity at 357 nm); blue (450 nm); and green (520 nm) (Bennett and Brown, 1985; White et al., 2003).
2. *Diurnal and crepuscular bees (not predicted, high frequency)*. The Hymenoptera have a highly conserved trichromatic visual system showing maximal sensitivity at 344 nm (UV), 436 nm (blue), and 544 nm (green) (Peitsch et al., 1992; Briscoe and Chittka, 2001).
3. *Diurnal swallowtail butterfly (not predicted, occasionally observed)*. We used the well‐studied tetrachromatic visual system of the swallowtail butterfly *Papilio xuthus* L. (Papilionidae), showing maximal sensitivities at 360 nm (UV), 400 nm (violet), 460 nm (blue), 520 nm (green), and 600 nm (red) (Arikawa, 2003; Koshitaka et al., 2008). It should be noted that *C. spinosa* has been reported as nectar source for butterflies (Pieridae and Papilionidae) in southern Spain and southern India (Fernández Haeger and Jordano Barbudo, 1986; Varshney, 1993; Venkata Ramana et al., 2004). The genus *Papilio* is present in the study area (Kantsa et al., 2018), but we have never observed them on *C. spinosa*; moreover, butterflies are expected to forage during the daytime after anthesis, when the flowers are senescent (Appendix S1, Figure S1), so their function as pollinators is doubtful.
4. *Diurnal hawkmoth (potentially predicted, not observed)*. We used the visual system of *Macroglossum stellatarum* L. [Sphingidae] (hummingbird hawkmoth), which occurs in the study area, although it has not been observed on *C. spinosa*. This hawkmoth shows maximal sensitivities at 349 nm (UV), 440 nm (blue), and 521 nm (green) (Telles et al., 2016).

We quantified the stimulation of visual receptors by the reflected light on each floral part using the function *vismodel()* of the package pavo 2.4.0 (Maia et al., 2019) in R version 4.0.2 (R Core Team, 2020). It should be highlighted that for all four models, we set the same illuminance level and background. Finally, in all models, we applied the von Kries transformation that repositions all points in color space such that the background is placed in the achromatic center (Maia et al., 2019).

For each visual system, we modeled the reflectance spectra in the respective color spaces by calculating the color loci in (a) the triangular spaces corresponding to the visual system of *Manduca sexta* (see Balkenius et al., 2004) or *Macroglossum stellatarum* (Telles et al., 2016), (b) the hexagonal space of the trichromatic vision of *Apis mellifera* L. [Apidae] (Chittka, 1992), and (c) the tetrahedral color space of the visual system of *Papilio xuthus* (Koshitaka et al., 2008). For these calculations we used the function *colspace()* in pavo 2.4.0.

For each floral part, we calculated color saturation according to each visual system. Saturation is a proxy of spectral purity, i.e., of the degree of how much grey and white light is mixed in with the pure color. Saturation, which may also be sometimes referred to as color contrast (van der Kooi et al., 2019; van der Kooi and Spaethe, 2022), was calculated as the polar coordinate denoting the distance of the loci from the center of the color space (*r*) (see also Shrestha et al., 2014; Kantsa et al., 2017). These values were extracted using function *colspace()* of pavo 2.4.0. To test the variation of color saturation among the three floral parts in each visual system, we applied one‐way repeated measures ANOVA, using the function *anova_test()* of the R package rstatix 0.7.1 (Kassambara, 2022), followed by pairwise post‐hoc t‐tests, using the arcsine‐transformed values of the dependent variable (proportion) to meet the assumptions of the parametric test.

Finally, we tested the degree to which the different floral parts can be correctly discriminated by different visual systems of pollinators, based on their perceived colors. For *A. mellifera* and *M. stellatarum*, high‐quality data from behavioral experiments (Dyer and Neumeyer, 2005; Telles et al., 2016) show that color discrimination can be expressed by a continuous, sigmoidal function dependent on chromatic difference expressed as geometric distance in a given color space (v. Helversen, 1972; Garcia et al., 2017). This suggests that, for short distances in the color space (i.e., similarly colored stimuli), pollinators choose at random, i.e., with an accuracy of 50%, between two stimuli as they cannot be discerned as being different from one another. With increasing color difference, accuracy increases until reaching a plateau. After this point, the probability of correct discrimination remains constant for increasing color differences until reaching the suprathreshold limit for easily discriminated colors (Telles et al., 2016; Garcia et al., 2017).

We calculated the probabilities for correct discrimination of the three floral parts by honeybees using a sigmoidal, three‐parameter logistic function described by Equation 1 (Garcia et al., 2017):

(1) $$h(V)=\frac{{Μ}_{o}K}{M_{o}+\left(K-\;M_{o}\right)\;\text{exp}(-\mathrm{rV})},$$

where the probability of an accurate discrimination (h) is expressed as a function of color dissimilarity (*V*), here measured as the Euclidean distance between two loci in the bee hexagon. The terms *Mo, K*, and *r* are constants unique to the observer and conditions being modelled. For *A. mellifera*, M_o_ = 0.492, denoting the lower asymptote; *K* = 1, denotes the upper asymptote; and *r =* 78.5, denotes the increment rate. Similarly, a four‐parameter sigmoidal function (Equation 2) can be used to predict the probability of accurate discrimination of color differences by *M. stellatarum*, modelled on the published results of wavelength discrimination experiments by this species (Telles et al., 2016):

(2) $$h(V)=\frac{{Μ}_{o}+(K-\;{Μ}_{o})}{1\;+\;\text{exp}(\frac{x_{\mathrm{mid}-\;V}}{r})},$$

where *V* represents color dissimilarity, measured as the Euclidean distance between two loci in the Maxwell triangle; M_o_ = 0.33; *K* = 1; *r =* 0.015; and *x*_mid_ = 0.143, denoting the scaling parameter, i.e., the position of the first inflection point on the *x*‐axis. In either case, by calculating the chromatic distance between two samples, we get the probability of correct discrimination by the animals, an approach which has been successfully applied in different pollinator groups (Garcia et al., 2017, 2021, 2022).

### Floral scent

Floral scent was sampled in the populations at Koudouroudia and Thermi in July 2011. In each population, nine healthy‐looking hermaphroditic flower buds from different individuals were selected in early evening before anthesis; the buds were first bagged with fine‐meshed tulle to exclude visitors. The first collection was carried out in vivo and in situ, for 90 min during nights with clear sky and calm weather between 20:00 and 21:30 (the sun sets at ca. 20:30). Additionally, we performed samplings at another two time points (repeated measures): night (01:00‐02:30), and dawn (05:30‐07:00) to examine temporal variability in scent emissions.

We employed dynamic headspace sampling, using PAS‐500 personal air samplers (Supelco, Bellefonte, Pennsylvania, USA) set at 200 mL · min^−1^ flow rate. The freshly opened flowers were enclosed in PET oven roasting bags with thickness of 12 μm (SANITAS, Sarantis Group, Maroussi, Greece) 10 min prior to sampling. Adsorbent traps contained 10 mg of Porapak® Q (80/100 mesh, Supelco), packed between two plugs of silane‐treated glass wool (Supelco) in a borosilicate glass Pasteur pipette (ø 7 mm). Additionally, during each sampling session, two ambient samples were collected from empty oven bags placed nearby to control for non‐floral contaminants.

We performed a separate sampling session in the population at Thermi to determine the intrafloral distribution of VOCs among the different floral parts: corolla, stamens, the module including the calyx and the gynoecium (which could not be separated without inflicting injury to floral tissues), and nectar. This experiment was inspired by our initial observation that the intense scent of *C. spinosa* emanated from the accumulated pollen grains inside the used headspace bags, indicating that one source of floral scent must be the anthers. To explore the intrafloral spatial variation of scent emissions, we sampled separately the petals (all four petals of the same flower were sampled together in the same headspace), stamens, and the calyx + gynoecium module (Appendix S1, Figure S2). We extracted the nectar accumulated in visitor‐excluded flowers during the first two hours after anthesis (until ca. 22:00) to sample for nectar‐released volatiles. We drained the nectar using micropipettes (Microcaps, Drummond Scientific, Broomall, Pennsylvania, USA) and transferred it to paper wicks (Whatman filter paper no.1, Cytiva, Marlborough, Massachusetts, USA), which were placed in the headspace bags (as outlined by Raguso, 2004) and sampled as described above. We sampled the headspace of intact dry paperwicks as control specimens.

Immediately after scent collection, the adsorbent traps were eluted with 300 μL of a 10:1 solution of hexane (puriss. p.a. – Merck, Hohenbrunn, Germany) and acetone (CHROMASOLV® for HPLC – Sigma‐Aldrich, Bellefonte, Pennsylvania, USA), recommended by Kaiser and Kraft (2001) to optimize elution of a full spectrum of polar to non‐polar volatiles. The eluates were stored in a freezer (–20°C) until chemical analysis. Before analysis, the scent samples were concentrated down to 50 mL with gaseous N_2_, and 1 ng of toluene (Fluka, Bellefonte, Pennsylvania, USA) was added as an internal standard to estimate emission rates (ER) in toluene equivalents per fresh mass of plant tissue (the sampled tissue was removed, placed in a portable cooler for ~1 h and was then weighed using a precision electronic scale). For the ER calculation we used the formula outlined by Svensson et al. (2005).

Scents were analysed on an Agilent 7890 A/5975 C GC/MS system (Agilent Technologies, Palo Alto, California, USA) using splitless injections at 240°C on a polar column (Agilent J&W DB‐WAX, length 30 m, ø 0.25 mm, film thickness 0.25 µm) and He as a carrier gas with a flow rate of 1 mL min^−1^. The GC oven was held initially at 40°C for 3 min and the temperature was increased at 10°C min^−1^ to 250°C for 5 min. The two eluents (hexane and acetone) were tested for contaminants using the same method; apart from some other traces, diacetone alcohol (CAS: 123‐42‐2) was the only abundant contaminant.

The Agilent MSD Productivity ChemStation software version E.02.01 (Agilent Technologies) was used to retrieve the GC/MS data, and AMDIS version 2.62 software for peak deconvolution combined with NIST 05 Mass Spectral Library version 2.0d (NIST Mass Spectrometry Data Center, Gaithersburg, Maryland, USA) to identify VOCs. Kováts Retention Indices were calculated for all the VOCs after analysis of an authentic alkane mix (C10‐C40; Sigma‐Aldrich) under the abovementioned chromatographic conditions, using the non‐isothermal equation of van den Dool and Kratz (Babushok, 2015). Published data on mass spectra and retention times and authentic standards were additionally used (from the literature and from NIST webbook; website https://webbook.nist.gov/). Whenever possible, we compared VOC retention times and mass spectra to those of authentic standards. Compounds were assigned to major biosynthetic classes based on the convention in the literature on floral volatiles (Knudsen et al., 2006).

To exclude that the emissions identified from the petals represented a “contamination” by the pollen from the dehiscing anthers during bud opening, we performed neutral red staining of the different floral parts for the macroscopic identification of scent‐producing tissues (Vogel and Hadacek, 2004); if only the anthers were stained, then emissions identified from petals could represent contaminations from copious pollen grains during dehiscence and bud opening. In this context, we let freshly opened hermaphrodite flowers soak into a 0.01% w/v aqueous solution of neutral red for 30 min, and then we observed the pattern of staining (Appendix S1, Figure S3).

To show the variability of the emissions across the three time points sampled (dusk, night, and dawn), we applied non‐metric multidimensional scaling (Bray‐Curtis distance) on the quantitative dataset using the function *metaMDS()* of the R package vegan version 2.5‐7 (Oksanen et al., 2020).

We tested for differences in the distribution of the VOCs among the different floral parts, by applying multivariate‐response linear models (MGLMs), in which the response variable was the entire quantitative matrix of the volatile distribution across samples (log+1 transformed), and the independent variable was the floral part. In total, three models were built corresponding to each one of the major biosynthetic pathways of *C. spinosa* scent (viz., nitrogenous compounds, benzenoids, and monoterpenes). The separate statistical analyses of the subsets (biosynthetic pathways) is deemed more conservative than testing the entire dataset, as it will occupy fewer degrees of freedom in each model tested, reducing the dimensionality of the data. Moreover, recent studies have indicated the utility of grouping compounds by biosynthetic class, resulting from common patterns of correlated data due to shared biosynthetic pathway flux and ecological/ecophysiological functionality (Kantsa et al., 2017; Kantsa et al., 2018; Eisen et al., 2022). For this, we used the function *manyglm()* (family: negative binomial) of the R package mvabund version 4.1.12 (Wang et al., 2012). The statistical significance of the fitted models was assessed with ANOVA (likelihood ratio tests) using 999 bootstrap iterations via PIT‐trap residual resampling. Univariate post hoc tests were then performed to determine which response variables (volatile compounds) varied significantly in their intrafloral spatial distribution (see also Kantsa et al., 2017).

Based on the total quantitative scent composition, we ran a hierarchical cluster analysis to determine the affinities among the volatile emissions of the different floral parts sampled. For this, we used Euclidian dissimilarity, and the Ward's minimum variance clustering method in the R function *hclust()*.

To test the quantitative variation of emission rates among the different plant parts, we applied ANOVA for each one of the three major biosynthetic classes, followed by post hoc Tukey's range tests.

### Visitor recordings

Hawkmoth visits are quick and can occur throughout the night, thus they can be easily missed. Therefore, motion‐activated night camera traps help to better understand nocturnal pollination (Steen, 2017; Johnson et al., 2020). In July‐August 2014, in the Mytilene population, we established a motion‐activated video monitoring system, consisting of a CCD (charge‐coupled device) camera with infrared LED illumination [Cat's Eye C15MS, 850 nm (IR wavelength), effective pixels: (PAL) 500(H) × 582(V)] and a standard 4.3 mm lens that was manually focused on flowers; the camera was connected to a mini digital video recorder (Cybereye DV‐100, resolution: 704 × 560) (for more details, see Steen, 2012). We monitored floral visitors over six nights between 23.07.2014 and 06.08.2014. In the early evening, before the buds opened, the camera was set to record one or two flowers of one individual of *C. spinosa* from 20:00 to 10:00 on the next day (Appendix S2). In total, we used three of the most robust plants, which we video‐recorded alternately during every night of the observation period. During all recordings, the weather was warm (T_min_ = 28.6° C, T_max_ = 32.8° C), the sky was clear, and the moon was transitioning from the waning crescent to the waxing gibbous phase. Floral visitors were identified with visual inspection of the recorded material. For each visitor taxon, visitation was calculated as *number of visits· flower* ^*−1*^ *· hour* ^*−1*^.

## RESULTS

### Floral reflectance and colorimetry

**Figure 1:**
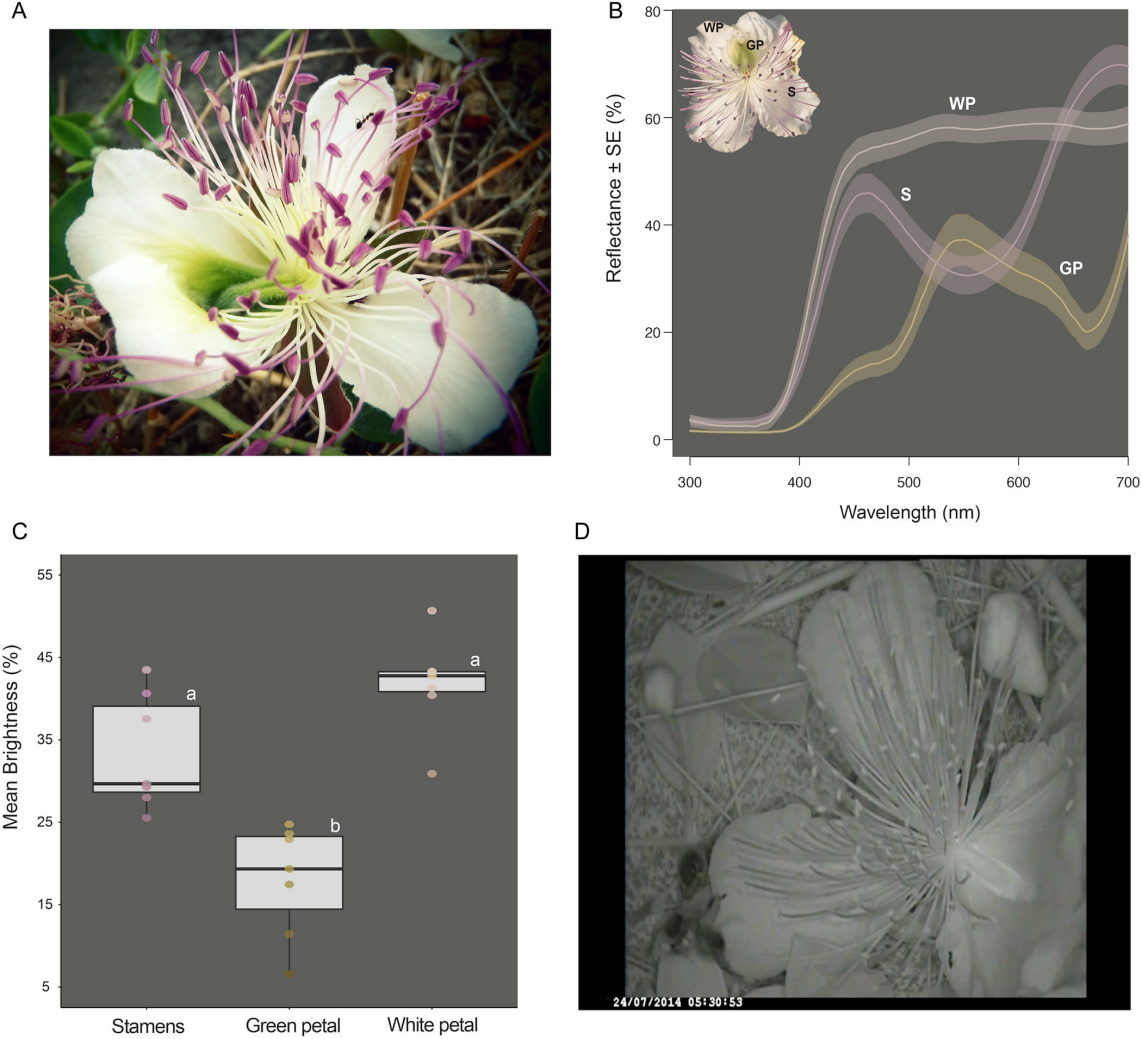
Intrafloral reflectance patterns of *Capparis spinosa*. (A) A male flower at the beginning of anthesis. (B) The reflectance spectra. For each floral part, the average spectra ± SE are shown. (C) The variation of the mean brightness of the reflectance spectra among the three floral parts. Statistical significance was estimated with a Friedman test followed by post‐hoc pairwise Wilcoxon signed‐rank tests (N = 7). (D) A snapshot of a flower as seen through the camera trap. WP: white petals, GP: green petals, S: stamens.

The reflectance spectra of the white petals, the green part of the dorsal petals covering the nectary, and the purple distal half of the stamens are shown in Figure 1. Interestingly, none of these parts reflects in the UV area (250‐400 nm) of the electromagnetic spectrum. The green area of the dorsal petals showed significantly lower levels of brightness compared with the other two floral parts measured (Friedman, Q = 12.3, *P* = 0.002) (Figure 1C). Moreover, the same area appears to exhibit stronger reflectance at the 850 nm (infrared area of the light spectrum), as seen through the CCD camera trap with infrared LED illumination, although we should note that the methodology used is not appropriate to capture thermal signatures (Figure 1D).

**Figure 2:**
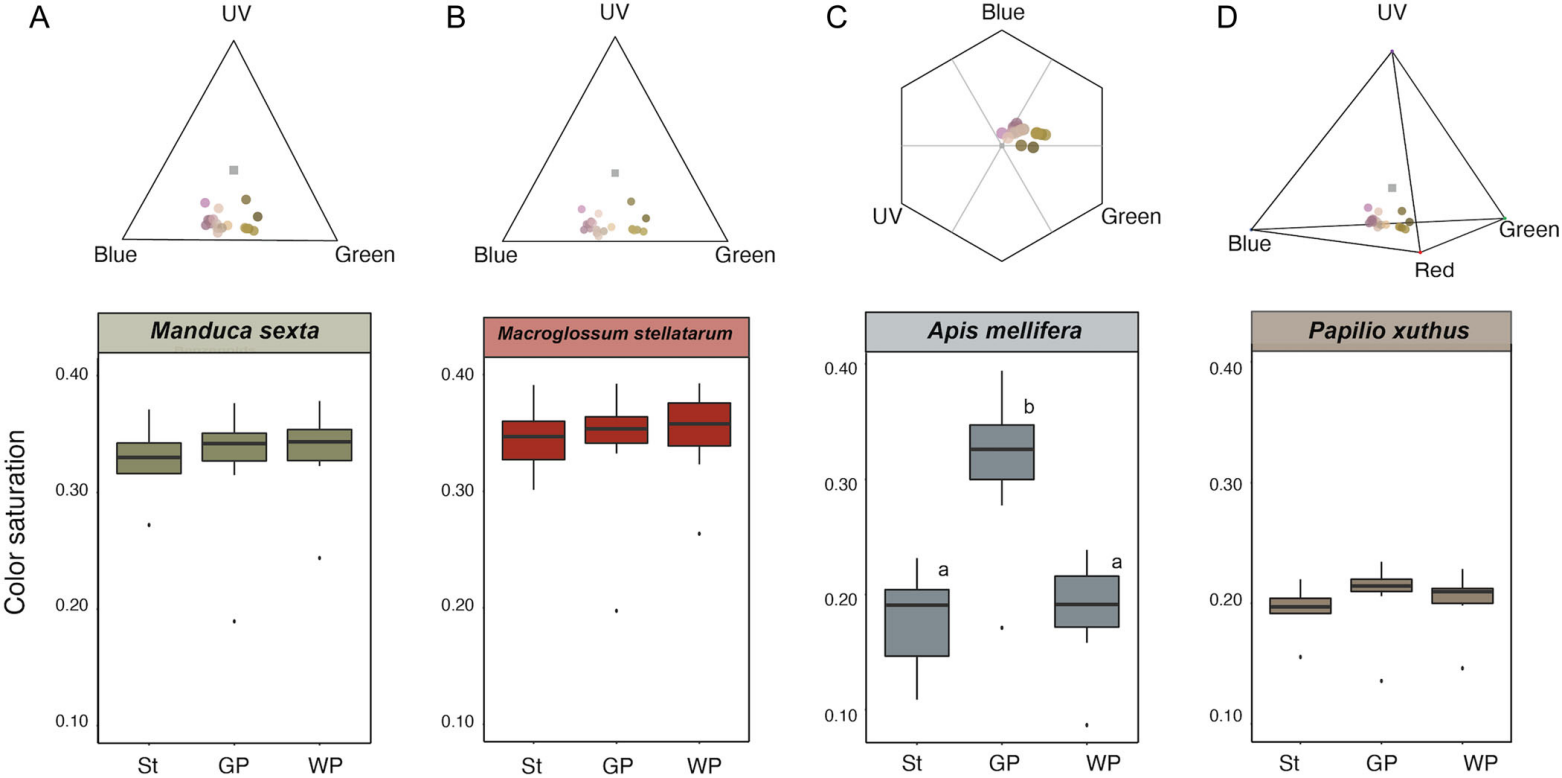
Intrafloral colorimetry in *Capparis spinosa*. The reflectance spectra (colored dots) mapped in the Maxwell triangle of the trichromatic color spaces of *Manduca sexta* (A) and *Macroglossum stellatarum* (B), honeybee color hexagon (C), and in the tetrahedral color space of *Papilio xuthus* (D) are shown. Grey dots represent the achromatic centers in each color space. The variation of color saturation among the three floral parts for the three visual systems are shown in the panels below (N = 7). Statistical significance was calculated with repeated measures ANOVA followed by pairwise t‐tests (saturation) (see also Appendix S1, Table S1). St: stamens; GP: green petals; WP: white petals.

The highest degree of color saturation (Appendix S1, Table S1; Figure 2) was found for the trichromatic visual systems of the hawkmoths (either *Manduca sexta* or *Macroglossum stellatarum*), in which all floral parts appeared equally color‐saturated. For the visual system of honeybees, the green part of the petals showed the highest degree of color saturation and differed significantly from the other flower parts. Finally, according to the visual system of swallowtail butterflies (*P. xuthus*), all floral parts showed equally low color saturation (Appendix S1, Table S1; Figure 2).

**Figure 3:**
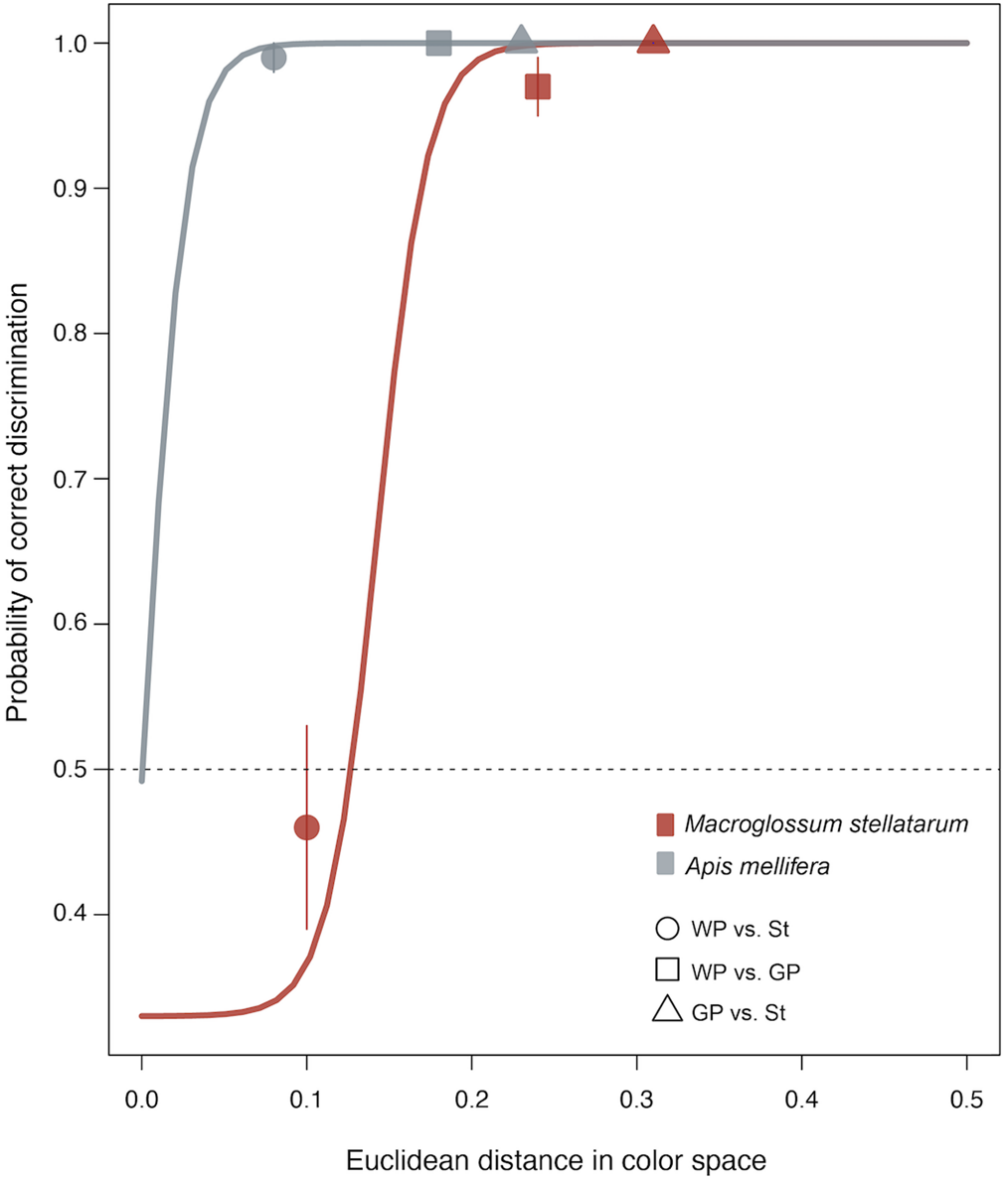
Mean probabilities of correct color discrimination of the different floral parts of *Capparis spinosa* by a hawkmoth (*Macroglossum stellatarum*) and a bee (*Apis mellifera*). For each visual system, the best‐fitting color discrimination sigmoidal functions are also shown. For the honeybee, the line represents the median value of 10,000 simulation discrimination curves following a three‐parameter logistic function (Garcia et al., 2017). For the hummingbird hawkmoth, the line represents the median value of 100,000 simulation curves following a four‐parameter logistic function fitting experimental data on wavelength discrimination (Telles et al., 2016). The dashed line denotes the probability of random color discrimination (*P* = 0.5). For all comparisons, N = 49. St: stamens; GP: green petals; WP: white petals.

The models of correct color discrimination showed that honeybees are predicted to accurately discriminate all floral parts of *C. spinosa* based on their color (Figure 3). In contrast, hummingbird hawkmoths (*M. stellatarum*) are predicted to accurately (P ~ 1.0) discriminate between white vs. green petals, and green petals vs. stamens, but to choose at random between white petals and stamens (*P =* 0.46 ± 0.07). as the colors of these organs cannot be discriminated from one another.

### Floral scent

**Figure 4:**
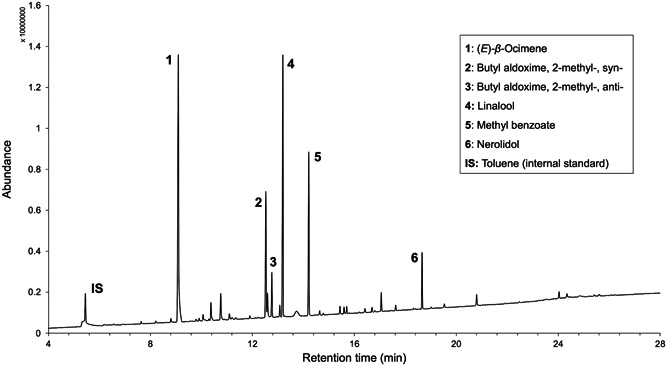
A typical gas chromatogram of the floral headspace of *Capparis spinosa*.

**Figure 5:**
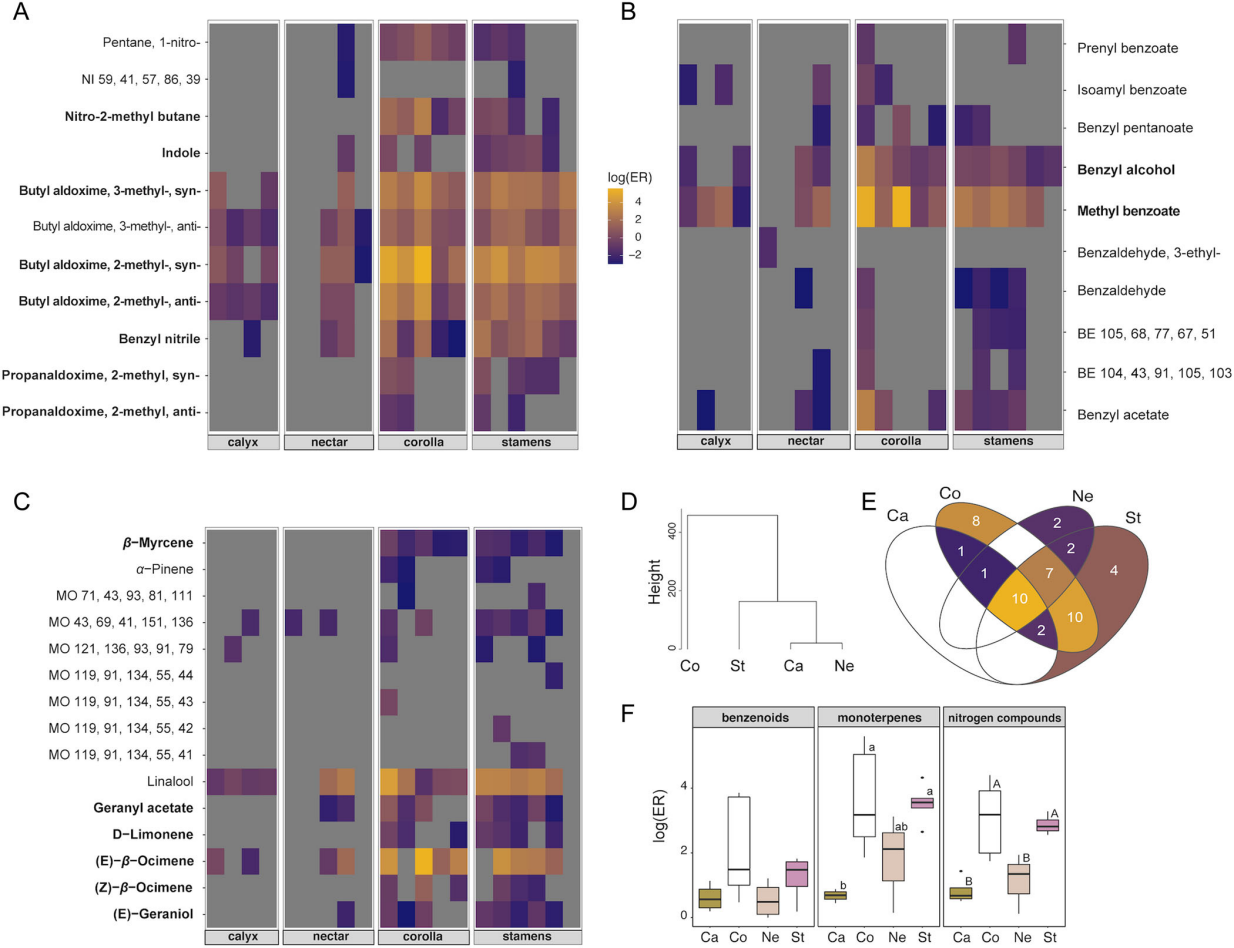
Intrafloral volatilomics of *Capparis spinosa*. (A–C) Heatmaps of the quantitative distribution of volatile compounds among the different floral parts and in the nectar of *C. 2q*. Each column of cells in the heatmap corresponds to a single sample. Separate heatmaps are presented for each one of the three major biosynthetic classes: (A) nitrogen compounds; (B) benzenoids and phenylpropanoids; and (C) monoterpenes. Compounds in bold denote statistically significant difference in their distribution among floral parts, according to the univariate post hoc tests following the multivariate‐response linear models (see Methods). (D) Hierarchical cluster analysis of the scent composition of the different floral parts. (E) Venn diagram showing the number of compounds shared by the floral parts. (F) Intrafloral differences in the emission rates (ER) of the different parts grouped by biosynthetic class. Statistical significance was acquired with ANOVA followed by Tukey's post hoc tests (Ca: Calyx + Gynoecium, N = 4; Co: Corolla, N = 5; Ne: Nectar, N = 5; St: Stamens, N = 6).

*Capparis spinosa* showed high floral volatile emission rates (Koudouroudia: 5.1 ± 1.8 μg fl^−1^ h^−1^; Thermi: 4.0 ± 1.0 μg fl^−1^ h^−1^), in which the major biosynthetic classes were monoterpenes, nitrogenous compounds, and benzenoids. In total, we identified 59 VOCs (Table 1, Figures 4, 5). In both populations studied, the floral scent emitted at dawn is highly variable compared with the early anthesis; especially the dusk emissions show the lowest level of variability, considering the quantitative composition of volatile blends (Appendix S1, Figure S4).

**Table 1:** The volatile organic compounds (VOCs) captured upon anthesis in the flowers of *Capparis spinosa* in two populations on Lesvos Island, Greece. For each VOC, the emission rate (ER) expressed in ng flower^−1^ hour^−1^ is given. Compounds marked with an asterisk have been identified based on comparison with authentic standards in the same chromatographic conditions. For the VOCs that could be assigned to a putative class but did not show a strong MS library match, the distinctive MS ion fragments, in descending order of abundance, are shown. KRI: Kováts Retention Index. For a comparison with published KRI under the same chromatographic conditions see Kantsa et al. (2017).

Biosynthetic class	KRI	VOC	Koudouroudia (N = 9)	Thermi (N = 9)
			Average ER (ng/f/h)	Average ER (ng/f/h)
**Aliphatics**	1102	Isoamyl acetate*	118.1 ± 70.1	30.9 ± 4.6
	1198	Isoamyl alcohol	28.0 ± 10.2	7.9 ± 2.5
	1328	Prenyl alcohol	3.2 ± 1.0	1.1 ± 0.8
	1949	AL 72, 43, 81, 71, 55	2.2 ± 0.8	0.5 ± 0.4
**Benzenoids**	1537	Benzaldehyde*	11.2 ± 3.1	4.8 ± 1.2
	1625	Methyl benzoate*	183.5 ± 95.8	344.0 ± 61.4
	1706	Benzyl acetate*	39.4 ± 23.9	18.6 ± 5.4
	1770	BE 104, 43, 91, 105, 103	3.1 ± 0.9	4.7 ± 1.6
	1771	BE 104, 105, 103, 65, 78	8.3 ± 2.9	7.9 ± 4.1
	1830	Benzyl alcohol	101.1 ± 49.7	44.8 ± 11.9
	1854	Benzyl pentanoate	4.0 ± 2.0	7.9 ± 2.6
	1890	Isoamyl benzoate*	24.8 ± 9.2	37.1 ± 14.3
	2007	BE 105, 68, 77, 67, 51	3.1 ± 0.8	4.9 ± 2.6
	2080	Prenyl benzoate	2.1 ± 0.8	4.7 ± 2.3
	2133	BE 104, 105, 77, 79, 103	2.0 ± 1.1	0.4 ± 0.4
	2852	Benzyl benzoate	13.0 ± 4.5	11.1 ± 4.4
**Irregular terpenes**	1311	(*E*)−4,8‐dimethyl‐1,3,7‐nonatriene	3.3 ± 1.3	—
	2130	Hexahydrofarnesyl acetone	2.3 ± 1.7	—
**Monoterpenes**	923	*α*‐pinene*	0.9 ± 0.5	2.5 ± 0.7
	1149	*β*‐Myrcene*	4.6 ± 1.9	16.4 ± 4.7
	1188	D‐Limonene*	4.1 ± 2.2	15.2 ± 4.5
	1229	(*Z*)*‐β*‐Οcimene	19.9 ± 6.9	18.7 ± 4.8
	1251	(*E*)*‐β*‐Οcimene	1418.7 ± 454.3	1083.9 ± 266.3
	1286	MO 121, 136, 93, 91, 79	0.7 ± 0.5	4.2 ± 1.3
	1383	MO 121, 136, 105, 79, 91	5.0 ± 1.8	3.3 ± 1.0
	1445	MO 119, 91, 134, 77, 79	2.6 ± 0.9	3.3 ± 1.2
	1458	MO 119, 91, 134, 55, 41	19.9 ± 5.7	14.5 ± 4.8
	1552	Linalool*	418.1 ± 216.6	932.6 ± 282.8
	1560	MO 71, 43, 93, 81, 111	2.5 ± 1.2	8.4 ± 2.9
	1701	MO 69, 41, 93, 43, 68	—	2.1 ± 0.7
	1724	Geranyl acetate*	9.7 ± 5.5	21.8 ± 5.4
	1757	MO 69, 41, 93, 67, 68	1.2 ± 0.6	5.0 ± 1.0
	1790	(*E*)‐Geraniol*	4.9 ± 1.3	10.4 ± 2.6
	1797	MO 43, 69, 41, 151, 136	1.2 ± 0.7	4.1 ± 1.0
	2268	MO 71, 43, 93, 55, 81	8.7 ± 2.3	—
**Nitrogenous compounds**	1054	Butanenitrile, 2‐methyl‐	1.2 ± 0.7	0.9 ± 0.4
	1098	Butanenitrile, 3‐methyl‐	50.6 ± 23.6	5.3 ± 1.5
	1323	Nitro‐2‐methyl butane	20.7 ± 8.9	24.3 ± 5.6
	1339	Nitro‐3‐methyl butane	76.7 ± 25.2	33.3 ± 6.3
	1399	Propanaldoxime, 2‐methyl, syn‐	5.7 ± 1.7	2.2 ± 1.5
	1417	Propanaldoxime, 2‐methyl, anti‐	2.0 ± 0.9	0.8 ± 0.5
	1504	Butyl aldoxime, 2‐methyl‐, syn‐	472.4 ± 135.1	444.7 ± 56.5
	1510	Butyl aldoxime, 3‐methyl‐, syn‐	947.5 ± 246.9	352.0 ± 70.8
	1522	Butyl aldoxime, 2‐methyl‐, anti‐	152.0 ± 45.7	161.6 ± 22.9
	1545	Butyl aldoxime, 3‐methyl‐, anti	547.4 ± 150.2	213.4 ± 48.4
	1592	Pentanal oxime	17.7 ± 16.9	—
	1619	NI 59, 41, 57, 86, 39	7.3 ± 2.4	9.8 ± 2.6
	1649	NI 59, 41, 57, 70, 86	—	5.8 ± 1.7
	1910	Benzyl nitrile	72.5 ± 27.5	64.8 ± 20.7
	2663	Indole*	22.6 ± 7.6	20.2 ± 7.5
**Sesquiterpenes**	1654	*β*‐Farnesene	113.8 ± 107.7	—
	1717	*α*‐Farnesene	9.7 ± 2.4	—
	2042	(*E*)‐Nerolidol*	114.1 ± 54.9	7.6 ± 5.7
	2360	SE 69, 41, 81, 93, 67	—	9.8 ± 3.0

The six identified nitrogenous aldoximes showed the highest combined emission rates (Koudouroudia: 41.6% of the total emissions in toluene equivalents; Thermi: 29.1%). (*E*)‐*β*‐ocimene represented >25% of the total emissions in both populations, followed by linalool (Koudouroudia: 8.2%; Thermi: 23.1%), and methyl benzoate (Koudouroudia: 3.6%; Thermi: 8.5%).

Regarding intrafloral volatilomics, there was a clear variation among the different floral parts and nectar for all three major biosynthetic classes (Figure 5A–C). The MGLM models showed that floral part was a significant source of variation for nitrogenous compounds (MGLM, Dev_3,16_ = 109.00, *P* = 0.001), benzenoids (MGLM, Dev_3,16_ = 35.84, *P* = 0.001), and monoterpenes (MGLM, Dev_3,16_ = 49.64, *P* = 0.001). The univariate post hoc tests identified several compounds that showed significant distribution patterns among the floral parts sampled and are shown in detail in Fig. 5A–C and in Appendix S1, Table S2.

According to hierarchical clustering, the petals showed the lowest volatilomic affinity to the other floral parts or to nectar (Figure 5D) and emitted the highest number of unique compounds (Figure 5E; Appendix S1, Table S3). The closest similarity was observed between the volatile blends of nectar and the calyx + gynoecium module. Ten compounds were spatially diffuse on the flower, i.e., they were shared among all floral parts and nectar, including mainly benzenoids (viz., benzyl acetate, methyl benzoate, benzyl alcohol, benzyl nitrile) and aldoximes (viz., 2‐methyl‐butyl aldoxime and 3‐methyl‐butyl aldoxime) followed by linalool, and one monoterpene (Figure 5A–C). Another ten compounds were identified in three out of the four floral parts sampled (Appendix S1, Table S5).

The highest floral emissions came from the anther‐bearing stamens and the corolla regarding all the major biosynthetic classes (Figure 5F). Overall, the lowest emissions came from the floral module calyx + gynoecium. Benzenoid emissions did not differ statistically among floral parts, although corolla emissions were highest per fresh mass (ANOVA, *F*_3,15_ = 2.66, *P* = 0.086). Nitrogenous compounds were emitted at higher rates from the corolla and the stamens, compared with calyx + gynoecium and nectar (ANOVA, *F*_3,14_ = 10.01, *P* < 0.001). A similar pattern was found for monoterpenes (ANOVA, *F*_3,13_ = 6.84, *P* = 0.005), although in the nectar their concentration was higher than nitrogenous compounds.

### Floral visitation

The highest overall visitation rate was observed around dusk, right at the beginning of anthesis (30.7 ± 11.7 visits fl^−1^ h^−1^), followed by dawn/morning (7.3 ± 1.7 visits fl^−1^ h^−1^). Nocturnal visits were observed only during three out of the six nights and were more infrequent than the other two time‐periods (0.2 ± 0.1 visits · fl^−1^ · h^−1^). The most frequent visits were by bees (Table 2; Appendix S2), which were active from the beginning of anthesis until ca. 21:30, and in the following morning from 05:30 to 10:00. Honeybees specifically sought the anthers with precise movements, without touching the stigma. This behavior was observed repeatedly, suggesting that honeybees should be considered as pollen thieves, rather than pollinators of *C. spinosa*. On the contrary, large carpenter bees, (viz. *Xylocopa olivieri* Lepelitier and *X. violacea* L. [Apidae]), which probed only for nectar, were potential pollinators since they swept pollen from the anthers with their large bodies and rough movements and could touch the stigma during a floral visit. In total, large bees showed an average visitation rate of one visit every 40 min per flower, either at dusk or after dawn.

**Table 2:** Floral visitors that were video recorded on *Capparis spinosa* in summer 2014 in Mytilene. Dusk: 20:00‐21:00; night: 21:00‐05:00; morning: 05:00‐10:00. Visitation rates are expressed in visits· fl^−1^· h^−1^ ± SE.

Family	Species	Time	Visitation rate (N = 6)
Apidae	*Apis mellifera*	Dusk	30.50 ± 11.70
		Night	—
		Morning	5.90 ± 1.61
	*Xylocopa olivieri*	Dusk	—
		Night	—
		Morning	0.33 ± 0.15
	*Xylocopa violacea*	Dusk	0.17 ± 0.17
		Night	—
		Morning	0.97 ± 0.32
Sphingidae	*Theretra alecto*	Dusk	—
		Night	0.17 ± 0.08
		Morning	0.03 ± 0.03
Syrphidae	*Eupeodes corollae*	Dusk	—
		Night	—
		Morning	0.03 ± 0.03

Nocturnal hawkmoths were considerably less frequent visitors of *C. spinosa* than bees. On average, the nocturnal visitation rate per flower corresponded to almost one visit every five hours. We recorded *Theretra alecto* L. (Sphingidae) in three out of the six nights of observations in 2014 (Table 2). The same species was observed occasionally in 2011 during scent samplings, along with the larger hawkmoth *Agrius convolvuli* L. (Sphingidae), always visiting between ca. 23:00 and 05:00. Hawkmoths clearly probed for nectar using their proboscides (Appendix S2), and pollen was accumulated on the abdomen and the proboscis, both of which contacted the stigma during flower visits.

Finally, in all populations, the flowers of *C. spinosa* were visited all night long by at least three morphospecies of ants (Appendix S2), which removed nectar by accessing the nectary underneath the corolla through the gaps between the sepals (see also Al‐Yousif, 2012), without touching any reproductive organ. In all populations, across the years, shield bugs *Palomena prasina* L. and *Eurydema* sp. (both Pentatomidae) formed large aggregations on plants (Appendix S1, Figure S5).

## DISCUSSION

Our detailed intrafloral analysis of sensory stimuli provides a framework for resolving the reproductive ecology and evolution of binary pollination systems, such as that observed for *C. spinosa*. We presented evidence for a clear trend towards hawkmoth attraction and identify the phenotypic components more likely to be attractive to bees.

### Brushes not for all heads

Brush flowers are often described as generalist flowers in terms of visitor spectrum, lacking clear adaptations for specific pollinator guilds (Kers, 2003; Willmer, 2011). Yet, empirical data from plant–pollinator communities from around the world showed that brush flowers can be unexpectedly specialized in their interactions (Olesen et al., 2007). In our opinion, affiliations between brush‐flowers and specific pollinator guilds should not be unquestionably excluded given that (a) cryptic specialization has indeed been shown, e.g., regarding the length of stamens, when pollen deposition is on the head and thorax of the hawkmoth (e.g., Moré et al., 2006), and (b) even when flower architecture is open to multiple pollinator guilds, filtering can still be realized using either the sensory component of the phenotype, i.e., color and/or scent (see also Kantsa et al., 2017) or the composition of nectar (Johnson et al., 2006). Even though these traits are quantitatively measurable and expressed with continuous variables, their inclusion in syndrome studies remains problematic, leading to conflicting or misleading conclusions (reviewed by Dellinger, 2020).

### One spot to guide them all?

It is well‐known that hawkmoths use color vision for foraging (Johnsen et al., 2006); however, the use of chromatic vs. achromatic cues in dim light conditions varies among species (van der Kooi and Kelber, 2022) and may be dependent upon ambient illuminance and background color (Kuenzinger et al., 2019). Hawkmoth‐pollinated flowers are usually white and do not reflect UV (White et al., 1994; Raguso et al., 2003a). This coloration (a) has been shown to promote associative learning (Kelber et al., 2003), (b) offers a stable achromatic contrast against the background by increasing signal reliability during the rapid transition from dusk to night (Johnsen et al., 2006), and (c) may indeed be innately attractive to naïve hawkmoths (Goyret and Yuan, 2015). Interestingly, UV‐absorbing white flowers are also well adapted for the trichromatic vision of bees and are very common in many environments (Dyer et al., 2021); in contrast, UV‐reflecting white flowers are difficult for bees to process, they are rare in nature, and are considered better adapted to bird pollination (Kevan et al., 1996; Lunau et al., 2011). Thus, the reflectance spectrum of the white petals of *C. spinosa* cannot be exclusively associated with hawkmoths.

We found a potential triple‐function nectar guide, including achromatic, chromatic, and mechanosensory information potentially relevant to both hawkmoths and bees. Nectar guides can serve more functions than attracting pollinators, e.g., discouraging illegitimate floral visits (Leonard et al., 2013), and here we show that the same multisensory guide may even target more than one pollinator class.

Firstly, the nectary access point was significantly less bright than the white petals or the stamens. We consider this contrast to serve as a potential achromatic nectar guide. In general, brightness is differentially important for diurnal pollinator attraction but it is innately important for at least two species of nocturnal hawkmoths (van der Kooi and Kelber, 2022), and it can predict proboscis placement, the alignment of the hawkmoth's body when feeding, and probing duration (Goyret, 2010).

Secondly, the surface of the green access point of the nectary is tomentose, covered with dense tangled woolly hairs (Appendix S1, Figures S1, S2). Given that no other part of the corolla is hairy, this surface provides distinct tactile cues to visitors. Interestingly, mechanosensory cues have been shown to be important for the correct alignment of the proboscis of hawkmoths, especially when the floral surfaces are large (Goyret and Raguso, 2006; Johnson et al., 2020), thus the indumentum could serve as a tactile nectar guide.

Thirdly, the access point of the nectary also represents a potential chromatic nectar guide for bees. This spot was the most chromatically saturated according to bee vision, reaching the same levels of saturation as in hawkmoth vision; in contrast, the white petals as well as the stamens showed much lower saturation in bee vision, as was true in swallowtail vision (Figure 2). Its potential role as bee‐oriented nectar guide is based on the known innate preference of bee species for surfaces with high spectral purity (Lunau, 1990; Rohde et al., 2013); besides, higher floral color saturation has been shown to predict nectariferous flowers and increased visitation by bees at the community level (Kantsa et al., 2017, 2018). A similar trend of a centripetal increase of saturation towards the access point of nectar has been recently found for the bee‐pollinated orchid *Cattleya walkeriana* Gardner (Aguiar et al., 2020).

The analysis of color discrimination revealed that hummingbird hawkmoths are not predicted to discriminate between white petals and stamens (Figure 3). Given that, in a frontal view of the flower, the white petals are mostly background for the stamens, the stamens are in fact camouflaged. Intriguingly, this is not the case for honeybees that, given the same viewing conditions, are predicted to accurately discriminate all floral parts from one another based on their color (Figure 3). Indeed, the known fine color discrimination of honeybees (v. Helversen, 1972; Giurfa, 2004; Dyer, 2012; Reser et al., 2012) obviously contributes to their precise movements and intensive exploitation of the pollen of *C. spinosa*, i.e., to their function as pollen thieves.

### A typically sphingophilous scent

During the transition from dusk to night, olfactory stimuli are critical for the location of flowers by hawkmoths (Balkenius et al., 2006; Goyret and Yuan, 2015; Haverkamp et al., 2016), especially in the absence of moonlight (Kuenzinger et al., 2019). This necessity has probably driven the establishment of a rather straightforward affiliation between specific combinations of floral VOCs and visitation by nocturnal hawkmoths, representing a notable point of reference in the controversial landscape of pollination syndromes (Knudsen and Tollsten, 1993; Raguso et al., 2003a, 2003b; Nielsen and Møller, 2015) and an association that has evolved more than once in the Mediterranean region (e.g., the hawkmoth‐pollinated *Pancratium maritimum* L. [Amaryllidaceae]) (Eisikowitch and Galil, 1971).

Our results revealed that the constitutive floral emissions of *C. spinosa* are typical of hawkmoth‐pollinated plants. Specifically, they match the “white‐floral” scent profile sensu Kaiser (1993), a term coined to describe the scents of hawkmoth‐pollinated white African orchids and general to night‐blooming, moth‐pollinated plants (tuberose, gardenia, jasmine, jonquils) used in the perfume industry. Such volatile blends are typically rich in acyclic terpenoids (e.g., linalool, ocimene, nerolidol), benzenoids, and nitrogenous compounds (e.g., indole, aldoximes, nitriles) (Levin et al., 2003; Raguso et al., 2003a, 2003b, 2007; Nielsen and Møller, 2015; Steen et al., 2019).

We found an increasing variability in the quantitative composition of floral scent from dusk to dawn (Appendix S1, Figure S4). This trend had been expected in the case that floral emissions were addressed primarily to crepuscular/nocturnal pollinators (i.e., which are active at the beginning of anthesis). A similar trend has been observed in the hawkmoth‐pollinated *Quisqualis indica* L. (Combretaceae), which, however, has a much longer anthesis time than our focal species (Yan et al., 2016). Between fertilization and complete senescence, several floral structures and their related functions shut down and/or volatiles may be metabolized (e.g., due to microbial activity) leading to increasingly variable VOC blends within the population (Schade et al., 2001; Theis and Raguso, 2005; Steenhuisen et al., 2010; Kutty et al., 2021).

The corolla and stamens form the major chemical modules within the flower of *C. spinosa*, probably providing the bulk of olfactory information to visitors. Specifically, they constitute the major sources of VOCs, they are statistically correlated with the most abundant compounds of the headspace (e.g., (*E)‐β‐*ocimene, 3‐methyl‐butyraldoxime and 2‐methyl‐butyraldoxime, methyl benzoate, benzyl alcohol etc.), and the corolla shows the highest chemodiversity in relation to the other floral parts (Figure 5). No pronounced contrasts were found between stamens and petals; therefore, we would rather not expect any short‐distance chemical guides for pollinators (see García et al., 2021). Besides, volatile compounds in rewards may as well have multiple functionalities involving mutualistic and/or antagonistic interactions (Dobson and Bergstrom, 2000; Raguso, 2004).

Interestingly, in *C. spinosa*, the six nitrogenous aldoximes, when combined, comprise 29.1 to 41.6% of the total volatile emissions. Aldoximes are derived from essential amino acids (valine, isoleucine, leucine, phenylalanine) (Bak et al., 2006) and, along with related nitriles and nitro‐compounds, they represent early metabolic products of the more complex biosynthetic conversion of amino acids to cyanogenic compounds and eventually glucosinolates (see Halkier and Gershenzon, 2006; Nielsen and Møller, 2015). Volatile aldoximes are emitted by sphingophilous flowers of various lineages in both temperate and tropical environments (Knudsen and Tollsten, 1993; Kaiser, 1993, 1994; Knudsen et al., 2006; Raguso et al., 2007; Eisen et al., 2022; Skogen et al., 2022), and in some cases, e.g., in *Lonicera periclymenum* L. (Caprifoliaceae), they can make up almost 50% of total emissions (Knudsen and Tollsten, 1993). Kaiser (1993) characterized aldoximes in hedonic terms as “scent modifiers”, providing the characteristic tone to the “basic” scent perceived by humans when smelling the scent of aromatic compounds in sphingophilous orchids. The role of aldoximes as non‐human semiochemicals seems to be context dependent, including (a) the potential attraction of noctuid moths to *Silene* (Caryophyllaceae) flowers (Dötterl et al., 2006), (b) electroantennographic sensitivity by common hawkmoths (e.g., *Hyles lineata* Fabricius (Sphingidae); Raguso et al., 1996), and (c) the indirect defense of *Populus* (Salicaceae) trees (Clavijo McCormick et al., 2014; Irmisch et al., 2014). Although behavioral assays have indeed revealed an innate preference of *H. lineata* hawkmoths for 3‐methyl‐butylaldoxime (Summers, 2013), and of *M. sexta* for both the 3‐methyl‐butyl‐ and the 2‐methyl‐butyl‐ isomers (Bisch‐Knaden et al., 2018), there are still open questions about aldoxime functionality as olfactory attractants in hawkmoth pollination. This is especially relevant to our case study, given that the floral emission rates of aldoximes in *C. spinosa* are enormous, implying a high metabolic cost of the chemical floral display (i.e., due to high amino acid consumption as biosynthetic precursors).

The monoterpenes (*E*)‐*β*‐ocimene and linalool also were abundant and spatially diffuse compounds in *C. spinosa*, including abundant representation in floral nectar (Figures 4, 5). These VOCs are ancestral floral metabolites among seed plants (Schiestl, 2010) and they are particularly abundant in hawkmoth‐pollinated flowers (e.g., Knudsen and Tollsten, 1993; Kawano et al., 1995; Raguso and Pichersky, 1995; Miyake et al., 1998; Levin et al., 2001; Jürgens et al., 2003; Dötterl et al., 2012; Steen et al., 2019; Eisen et al., 2022; Skogen et al., 2022). Linalool can be positively associated with various floral visitors, e.g., Apidae bees, ants, beetles, etc. (e.g., Ikeda et al., 1993; Theis, 2006; Dötterl et al., 2012; Schiestl and Glaser, 2012; Kantsa et al., 2019). Even though floral blends containing linalool can be innately attractive to *M. sexta* (Riffell et al., 2009), the broader association between linalool (including its specific enantiomers) and nocturnal hawkmoths appears to be more complicated. For example, in behavioral experiments using flower‐naïve *M. sexta* or *H. lineata*, this compound was found to be innately neutral as a single odorant (Adam et al., 2021; Balbuena et al., 2022, respectively), whereas it can be learned by *M. sexta* in conditioning experiments (Gage et al., 2013). Finally, the experimental addition of linalool to flowers of *Oenothera harringtonii* W.L. Wagner (Onagraceae) reduces oviposition by *H. lineata*, which represents a pollinating herbivore for this plant (Balbuena et al., 2022). Consequently, the functional role of this compound in *C. spinosa* should not be taken a priori as a hawkmoth attractant, and additional/alternative explanations, such as pleiotropy, behavioral redundancy or defense against antagonists should be considered. The finding that floral linalool repels nectar‐thieving ants in central European flower communities (Junker and Blüthgen, 2008; Schiestl and Glaser, 2012) illustrates the importance of considering alternative functional roles for widespread floral volatiles.

Although we have described the floral scent of *C. spinosa* as typically sphingophilous, it is obviously not repellent to other pollinator guilds, nor to sap‐sucking shield bugs (Appendix S1, Figure S5) or nectar‐thieving ants (Al‐Yousif, 2012). The latter generates some interesting questions. Given the massive floral emissions, the persistent activity of the ants (throughout the night) indicates that the flowers of *C. spinosa* either are chemically defenseless against nectar theft or they may provide nectar as a reward to ants, possibly in some mutualistic context. In the first case, the lack of chemical defenses against native ants could have interesting biogeographic implications, given that the native floral scents generally fail to repel invasive, but not native ants (Junker et al., 2011). On the other hand, the exploitation of floral nectar by ants in exchange for defense is rare compared with the exploitation of extrafloral nectaries (EFNs) (Lach, 2008; Bleil et al., 2011). Within the genus *Capparis*, there are known species both with (e.g., *C. retusa* Griseb., *C. salicifolia* Griseb., *C. ecuadorica* Iltis) (Iltis, 1978; Farji Brener et al., 1992) and without EFNs (e.g., *C. spinosa* L., *C. indica* (L.) Druce, *C. odoratissima* Jacq., *C. buwaldae* M. Jacobs) (Haber et al., 1981; Maschwitz et al., 1996), all of which have known relationships with ants. Thus, the exploration of the floral volatilome and ants across *Capparis* spp. could help to address current challenges in understanding nectar‐mediated ant–plant ecology (see Nepi et al., 2018).

### Where are the hawkmoths?

Although *C. spinosa* represents a constant nectar resource for the vagrant hawkmoths crossing the Mediterranean Basin in the driest season of the year (Petanidou et al., 1996), hawkmoths are consistently rare visitors to this plant. We found that, on Lesvos Island, the potentially pollinating bees (*Xylocopa olivieri* and *X. violacea*) showed an 8‐fold higher visitation rate than hawkmoths (Table 2), corroborating previous research spanning four decades (Eisikowitch et al., 1986; Petanidou et al., 1996; Dafni, 1997; Zhang and Tan, 2009). Ηawkmoth visitors (*Hyles lineata* subsp. *livornica* Esper and *Agrius convolvuli*) are by far less frequent or entirely absent from many populations (for Greece, see Appendix S1, Table S4) (Eisikowitch et al., 1986; Dafni et al., 1987; Petanidou, 1991; Petanidou et al., 1996). In Turpan, NW China, Zhang and Tan (2009) found that large carpenter bees (*Xylocopa valga* Gerstaeker and *X. sinensis* Wu [Apidae]) were the principal floral visitors of *C. spinosa* and concluded that the only hawkmoth species observed (*A. convolvuli*) was not a pollinator because its body did not touch the anthers or stigma. We disagree with this conclusion for *C. spinosa* because hawkmoths can finely pollinate flowers with the pollen that they pick up with their proboscides, even in short‐tubed flowers (Kislev et al., 1972; Grant and Grant, 1983; Haber and Frankie, 1989; Willmott and Búrquez, 1996)—but see Peter et al. (2009). Overall, although the above studies did not discuss any shortfalls or biases of their methodologies, they underlined an interesting fact, i.e., *hawkmoths are unpredictable*, and they raised a puzzling question, i.e., *where are the hawkmoths?*

In cool‐temperate regions, hawkmoths are considered as *“largely absent”* pollinators (Endress, 1994) principally due to climate: the lower the night temperature is, the fewer hawkmoths fly (Baker, 1961). For example, along altitudinal clines, hawkmoth flight activity is restricted by low temperatures, thus other pollinator guilds replace them (Cruden et al., 1976; Miller, 1981; Amorim et al., 2014). However, observations of hawkmoth pollination can be rare also in the tropics (Nilsson et al., 1985); in Table 3, we present examples of published surveys, in which hawkmoths are reported as rare or absent for successive flowering seasons even in tropical lowlands, where temperature is not restrictive. Thus, the unpredictability of hawkmoths as pollinators is not exclusively linked their thermal biology. Alternative reasons could include:

- Hawkmoths are not central place foragers and do not defend territories – individuals are vagrant, giving rise to “big nights” of high visitation followed by long periods of absence (e.g., Artz et al., 2010)
- Yearly variation in population cycles due to enemies and/or short‐term climatic fluctuations (e.g., temporary aridity) (e.g., Grant, 1937; Willmott and Búrquez, 1996)
- Yearly variation in use of floral resources including non‐sphingophilous species that may be more abundant in that year (Kislev et al., 1972; Haber and Frankie, 1989)
- Sampling/observation shortfalls; the use of camera traps has only recently begun to be employed (Johnson et al., 2020)
- Differential visual attraction of the vagrant individuals to the same plant species depending on celestial conditions (e.g., lunar phase, overcast skies, artificial lights, etc.) (Kuenzinger et al., 2019; Deora et al., 2021).

**Table 3:** Examples of studies in which hawkmoth pollinators be absent for entire seasons or show an unexpectedly low visitation, although they had been considered as the principal pollinators of the focal plant species or in the respective communities. Exact study locations that were within (sub‐)tropical climatic zones are written in bold.

Family	Taxon/community	Location	Reference	Special notes
Acanthaceae	*Aphelandra acanthus*	**Ecuador** ^†^	Muchhala et al. (2009)	Hawkmoths very rare, but carrying large pollen loads
Agavaceae	*Manfreda virginica*	Tennessee	Groman and Pellmyr (1999)	
Amaryllidaceae	*Griffinia gardneriana*	**Brazil**	Albuquerque‐Lima et al. (2020)	Low visitation
Cactaceae	*Cereus fernambucensis*	**Brazil**	Locatelli and Machado (1999)	Hawkmoths present only during two of the six months of the flowering period
	*Echinopsis ancistrophora*	**Argentina** ^†^	Schlumpberger et al. (2009)	
	*Echinopsis chiloensis*	Chile	Walter (2010)	Low visitation
	*Peniocereus* spp.	Sonoran Desert	Raguso et al. (2003a)	
	*Selenicereus wittii*	**Brazil**	Barthlott et al. (1997)	Pollinators never observed in nature
Campanulaceae	*Brighamia insignis*	**Hawai'i**	Walsh et al. (2019)	Pollen limited plant population
Capparaceae	*Capparis indica* *	**Costa Rica**	Haber and Frankie (1982)	
	*Capparis ovata* *	Israel	Dafni et al. (1987), Eisikowitch et al. (1986)	
	*Capparis spinosa* *	Israel	Dafni et al. (1987), Eisikowitch et al. (1986)	
	*Capparis spinosa* *	Greece	Petanidou (1991)	
	*Capparis spinosa* *	Greece	Petanidou et al. (1996)	
Caryophyllaceae	*Viscaria vulgaris*	Sweden	Kwak and Jennersten (1986)	Hawkmoths usually 10% of the visitors
	*Silene vulgaris*	Sweden	Pettersson (1991)	High variation of abundance among three years
Cleomaceae	*Cleome gynandra* *	**Tanzania**	Werth (1942)	
	*Cleome lutea* *	Utah	Cane (2008)	
	*Cleome serrulatα* *	Utah	Cane (2008)	
	*Cleome spinosa* *	**Brazil** ^†^	Machado et al. (2006)	
Fabaceae	*Inga sessilis* *	**Brazil** ^†^	Amorim et al. (2013)	
Malvaceae	*Luehea* spp.*	**Costa Rica**	Haber and Frankie (1982)	
Onagraceae	*Clarkia breweri*	California	Miller et al. (2014)	Hawkmoths absent in the lowland populations
	*Oenothera cespitosa*	Utah^†^	Artz et al. (2010)	High variability across years
Orchidaceae	*Angraecum striatum*	**Réunion** ^†^	Micheneau et al. (2006)	
	*Habenaria epipactidea*	South Africa	Peter et al. (2009)	Low visitation
	*Habenaria johannensis*	**Brazil** ^†^	Moré et al. (2012)	
	*Platanthera chlorantha*	Norway	Steen (2012)	Low visitation
Ranunculaceae	*Aquilegia coerulea*	Colorado	Miller (1981)	Not observed for entire seasons
Rubiaceae	*Isertia laevis*	**Ecuador** ^†^	Wolff et al. (2003)	Low visitation – Pollen limited plant
	*Oxyanthus pyriformis*	South Africa	Johnson et al. (2004)	Rarely observed over four seasons
	*Psychotria homalosperma*	Japan	Watanabe et al. (2018)	Low visitation
	*Randia itatiaiae*	**Brazil** ^†^	De Avila Jr and Freitas (2011)	No visits for two flowering seasons
Solanaceae	*Petunia axillaris*	**Uruguay**	Dell'Olivo and Kuhlemeier (2013)	Rare for years
	*Jaborosa integrifolia*	Argentina	Vesprini and Galetto (2000)	Low visitation – Pollen limited plant
	*Jaborosa runcinata*	Argentina^†^	Moré et al. (2020)	Hawkmoths absent from several populations
	*Jaborosa odonelliana*	**Argentina** ^†^	Moré et al. (2020)	
	*Schizanthus* spp.	Chile	Pérez et al. (2006)	
Verbenaceae	*Citharexylum myrianthum*	**Brazil**	Rocca and Sazima (2006)	Low visitation – Pollen limited plant
NA	Pampas community	**Brazil**	Lautenschleger et al. (2021)	Not observed for six successive months
	Highland Atlantic rain forest	**Brazil** ^†^	Amorim et al. (2014)	
	Cerrado community	**Brazil** ^†^	Oliveira et al. (2004)	

* Brush flowers.

^†^ Populations studied at >700 m a.s.l.

### Does functional transition require a phenotypic transition?

Our findings disclose two paradoxes about the reproductive ecology of *C. spinosa*. First, although we highlight a binary pollination system (bees/hawkmoths), the floral phenotype is clearly biased towards moths, particularly regarding the timing of anthesis and volatile emissions. Obviously, no “pro‐bee” chemical adaptations were necessary for establishing carpenter bee visitation (Castellanos et al., 2004), as long as anthesis partly overlapped with the bees' foraging activity (see also Artz et al., 2010). Moreover, regarding visual cues, we showed that bees already possess the fine color vision capacity to perfectly discriminate between floral parts, which also facilitates honeybees' pollen‐thieving behavior (Figure 3, Appendix S2). Evidently, by utilizing similar visual capabilities, carpenter bees and honeybees can access flowers at dusk and dawn, with opposing fitness consequences for the plant.

Visitation to hawkmoth‐pollinated flowers by honeybees and carpenter bees is relatively common, both in the Mediterranean region (Eisikowitch and Lazar, 1987) and in similar habitats elsewhere (Barthell and Knops, 1997). Here we would like to point out that the bees visiting *C. spinosa* are known as trophic generalists. Moreover, at least in the Mediterranean, *C. spinosa* has very few co‐flowering competitors for pollinators, due to (a) summertime flowering, and (b) nocturnal anthesis. Thus, it could be hypothesized that a phenotypic transition should not be necessary, if the metabolically costly floral display could be maintained, a fact that brings us to the second paradox, i.e., the over‐stated floral advertisement, with particularly high N‐emissions, coupled with copious nectar rewards, appears to target a putative pollinator class that is rare or inconsistent.

Fitness trade‐offs are often key for understanding floral specialization, especially in mixed pollination systems (Aigner, 2001; Armbruster, 2017). Hawkmoths can be highly effective pollinators (especially for self‐incompatible and/or rare species) as they carry large pollen loads and promote outcrossing by travelling long distances (Haber and Frankie, 1989; Brunet and Sweet, 2006; Cruz‐Neto et al., 2011). For example, in Colorado, USA, pollination by *H. lineata* has been shown to prevent genetic drift among fragmented populations of *Oenothera harringtonii*, a rare endemic plant (Skogen et al., 2019). In Israel, Dafni et al. (1987) calculated similar overall pollination efficiencies (fruit set) between bees and sphingid moths for *C. spinosa*, although the bees visited the flowers at a 3‐fold higher rate. Thus, we hypothesize that *C. spinosa* maintains a binary pollination system, in which even occasional hawkmoth visitation is beneficial, and the frequent pollination by carpenter bees probably provides reproductive assurance; similar cases include *Aquilegia coerulea* E. James (Ranunculaceae) in the Rocky Mountains (Brunet and Sweet, 2006), *Oenothera elata* Kunth (Onagraceae) in coastal California (Barthell and Knops, 1997), and *Lonicera japonica* Thunb. (Caprifoliaceae) in lowland environments in Japan (Miyake and Yahara, 1998). Overall, we suggest that the case of *C. spinosa* fits the finding of the global meta‐analysis of Rosas‐Guerrero et al. (2014): in most cases in which floral phenotype failed to predict the most‐efficient pollinator, the pollinator predicted by the “syndrome” was still present but played a secondary role; thus, the floral phenotype possibly indicated the ancestral pollination system.

The strong selective imprint of hawkmoths on the flowers of *C. spinosa* requires sufficiency of amino acids (for N‐volatiles) and water (for nectar). We suggest that the conspicuous but ephemeral flowers of *C. spinosa* are not resource‐limited, probably owing to two important ecophysiological adaptations that secure resource sufficiency. First, the plant fixes its own N_2_ (Andrade et al., 1997), and its secondary metabolism is dominated by the pathways that generate glucosinolates, precisely the same pathways that lead (earlier, as intermediates) to aldoximes and other nitrogenous VOCs (Nielsen and Møller, 2015). To our knowledge, this is the first reported case of high nitrogenous floral emissions in a N_2_‐fixing plant (c.f. only trace emissions of indole and similar VOCs in cowpeas and acacias [both Fabaceae; Willmer et al., 2009; Andargie et al., 2014]). Second, its particularly deep root system, allowing permanent access to water (Rhizopoulou et al., 1997; Rhizopoulou and Kapolas, 2015), probably secures nectar production at the peak of the Mediterranean summer (Petanidou et al., 1996). Consequently, even if bee‐pollination is a derived state, no phenotypic changes have been necessary to attract carpenter bees, as long as anthesis temporally overlaps with their activity. We suggest that in similar cases, in which costly flowers seem paradoxical in relation to pollinator visitation (e.g., Morse and Fritz, 1983), eco‐physiological and reproductive traits may have to be assessed in unison to reach appropriate conclusions (cf. Borges et al., 2016).

## CONCLUSIONS

Although the flowers of *C. spinosa* are visited by pollinating carpenter bees at an 8‐fold higher rate than hawkmoths, our intrafloral analysis of sensory cues revealed that: (a) floral scent is typically sphingophilous, (b) petals and anthers are the main sources of VOCs, and (c) sensory/ecological flexibility is implied by reflectance and color patterns. Our findings strongly suggest that *C. spinosa* has evolved in tight connection with hawkmoths, without evolving concomitant traits that exclude visitation by large bees.

Intriguingly, the observed inconsistency between flower visitation and phenotype has been maintained owing to the lack of resource limitation for water and nitrogen, representing a unique case in literature. We believe that it is plausible that *C. spinosa* evolved indeed in a (sub‐)tropical semi‐arid area, where nocturnal hawkmoths were key pollinating agents. Currently, the adaptive peak for *C. spinosa* would be regular pollination by large bees, enhanced by irregular pollination (perhaps with greater outcrossing potential) by hawkmoths.

Given its ambiguities, the natural history of *C. spinosa* is not simple to track. However, the genus *Capparis* provides a valuable case study of comparative floral ecology and evolution, given its vast geographical distribution and the multiple floral morphs and pollination systems represented therein, indicating a high degree of evolutionary plasticity.

## AUTHOR CONTRIBUTIONS

A.K. and T.P. conceived the idea; A.K. collected the data; A.K., J.E.G., R.A.R., A.G.D., R.S., and T.T. contributed analysis tools; A.K. and J.E.G. performed analyses; A.K. wrote the first draft; all authors contributed to subsequent drafts.

## Supporting information


**Appendix S1**. Figures S1–S5; Tables S1–S5.

**Appendix S2**. Video “Floral visitors of *Capparis spinosa* on Lesvos Island, Greece.”

## Data Availability

All data used to generate the results are provided in the manuscript and the supporting information.
