# Supplementary material for: Intrafloral patterns of color and scent in Capparis spinosa L. and the ghosts of its selection past
Source: Am J Bot. 2022 Dec 15;110(1):e16098. doi: 10.1002/ajb2.16098 (PMC10108209; doi:10.1002/ajb2.16098)
Supplement: Supplementary file 1 — Appendix S1. Figures S1–S5; Tables S1–S5. [file AJB2-110-0-s002.docx]

# **Appendix S1**

Intrafloral patterns of color and scent in *Capparis spinosa* L. and the ghosts of its selection past

Aphrodite Kantsa, Jair E. Garcia, Robert A. Raguso, Adrian G. Dyer, Ronny Steen, Thomas Tscheulin, Theodora Petanidou

| 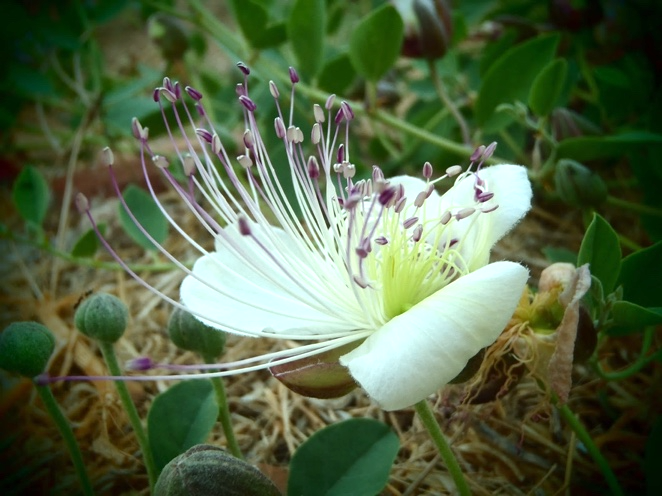 |
| --- |
| 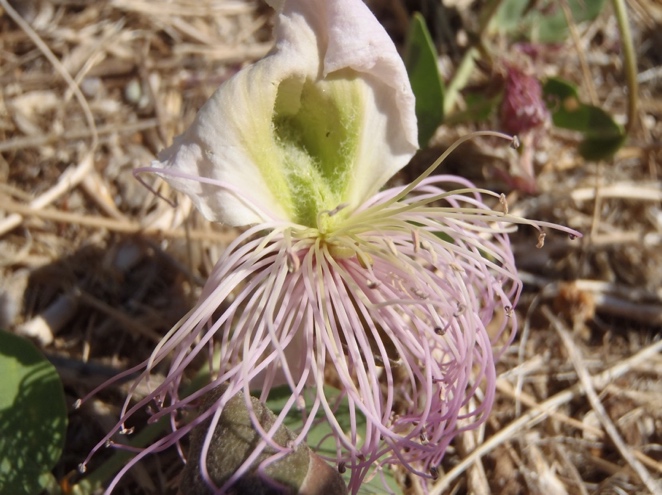 |

Figure S1. Male flowers of *Capparis spinosa* at the 20:30 (above) and at 09:20 on the following morning (below) in the population of Thermi, Lesvos Island. Photos: A. Kantsa.

| 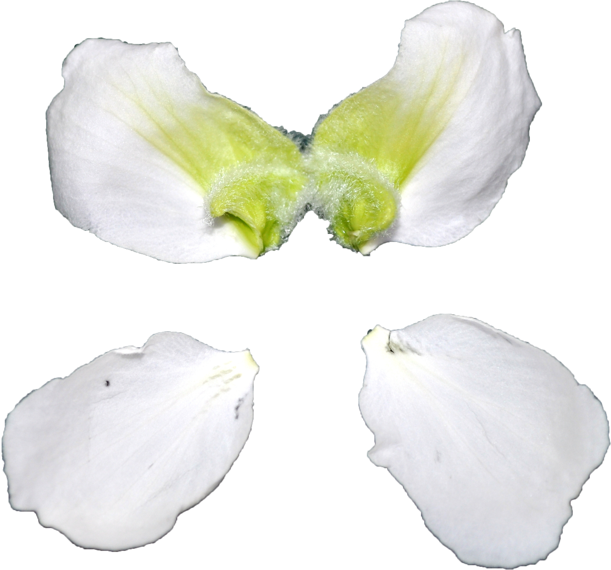 | |
| --- | --- |
| 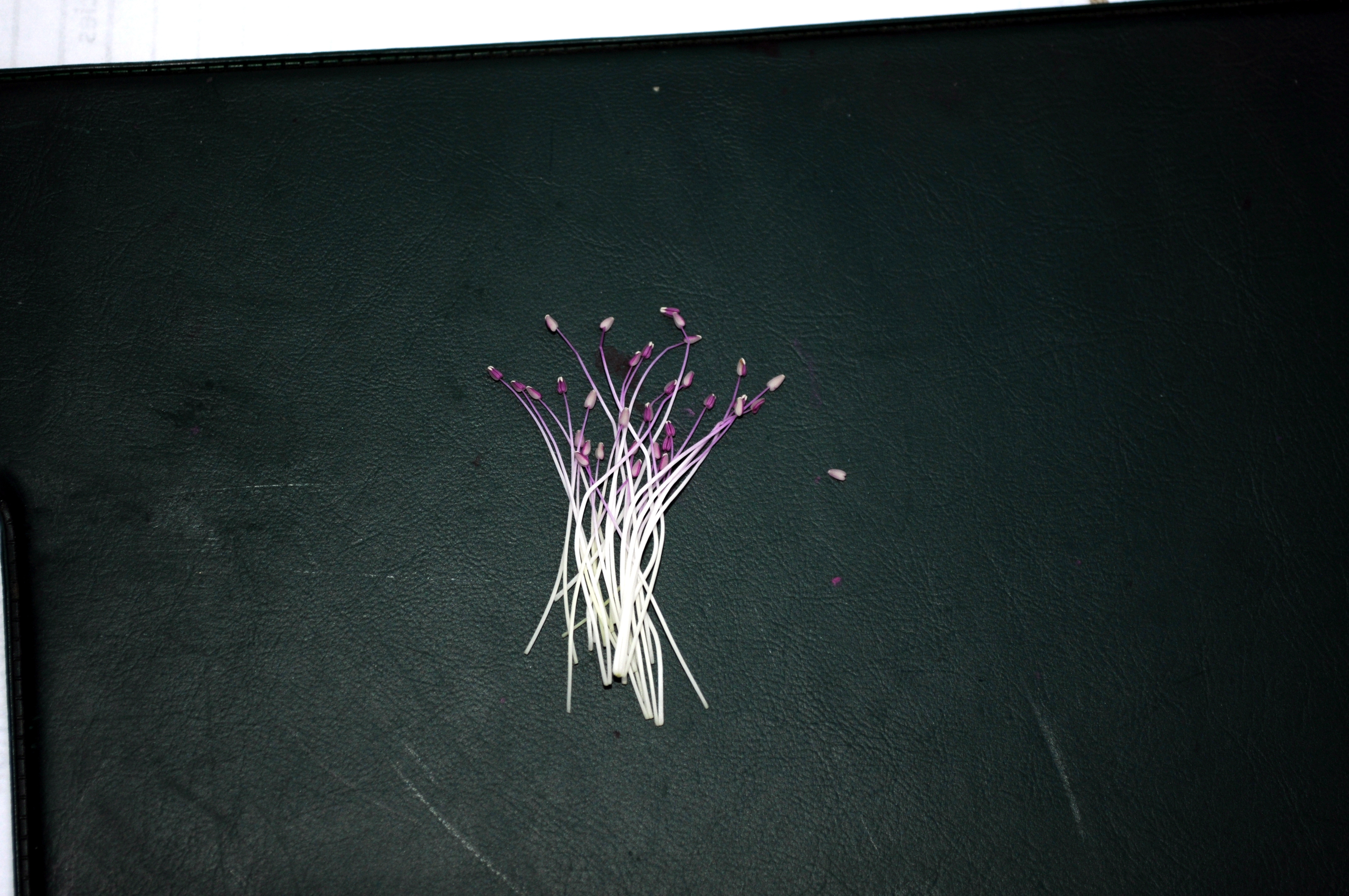 |  |

Figure S2. The floral parts of *Capparis spinosa* as used in the separate headspace samplings. Above: petals. Below left: stamens. Below right: the morphological module of calyx and gynoecium (the blue arrow indicates the position of the nectary). Photo: A. Kantsa.

| 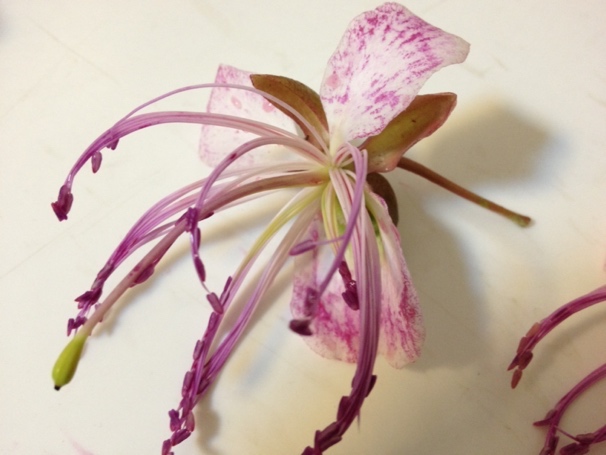 | 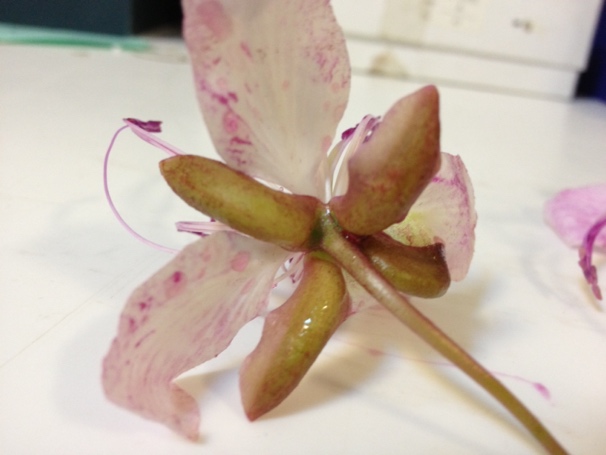 |
| --- | --- |
| 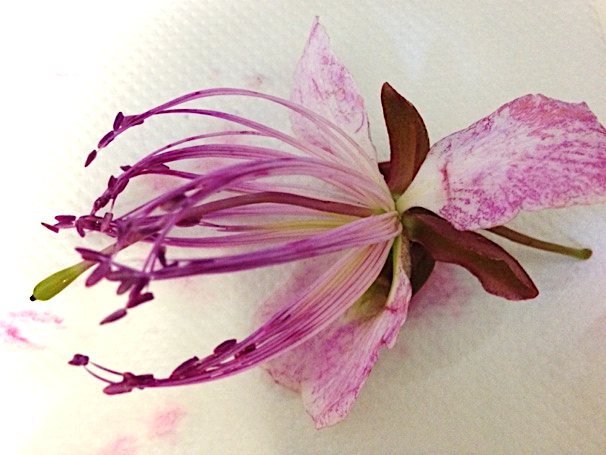 | 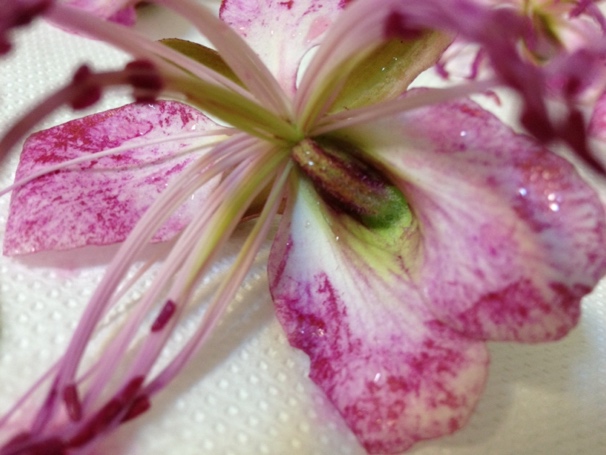 |

Figure S3. Flowers of *Capparis spinosa* that have been stained with neutral red. Photos: A. Kantsa.

Table S1. Results of the one-way repeated measures ANOVA testing for variation of color saturation among the three floral parts of *Capparis spinosa.* P-values denoting significance are written in bold.

| **Visual system** | **df** | ***F*** | **P-value** |
| --- | --- | --- | --- |
| *Manduca sexta* | 2,12 | 0.1 | 0.937 |
| *Macroglossum stellatarum* | 2,12 | 0.1 | 0.875 |
| *Apis mellifera* | 2,12 | 13.6 | **<0.001** |
| *Papilio xuthus* | 2,12 | 0.3 | 0.742 |


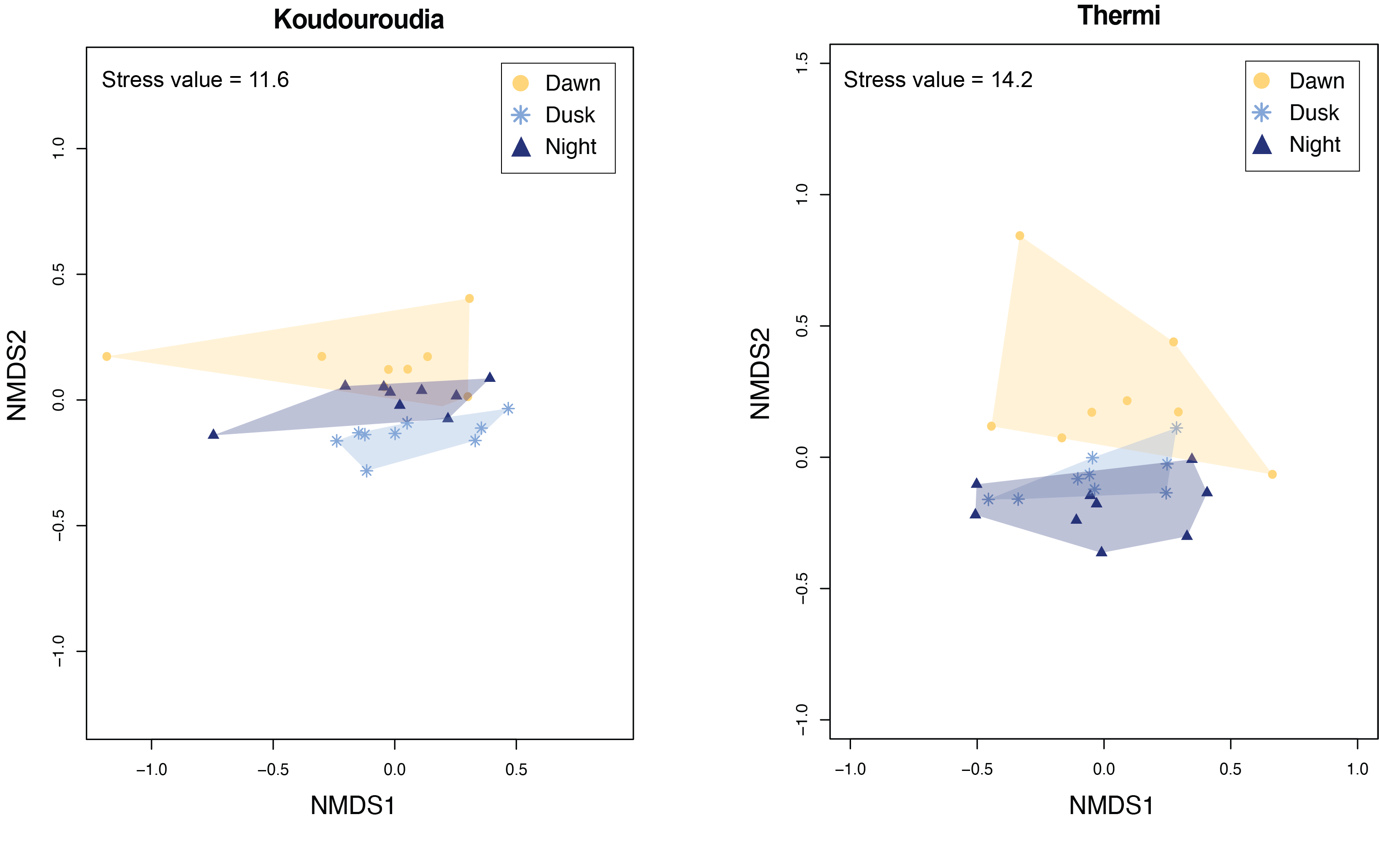


Figure S4. Non-metric multidimensional scaling plot of the quantitative composition (Bray-Curtis dissimilarity) of the floral emissions of *Capparis spinosa* in the two sampling sites on Lesvos Island, across the three time points sampled

Table S2. Results of the post hoc univariate tests following the MGLMs, including only the compounds showing significantly different distribution (P < 0.050) among the floral parts of *Capparis spinosa*. Statistics were acquired with ANOVA (likelihood ratio tests) using 999 bootstrap iterations via PIT-trap residual resampling.

| **Biosynthetic class** | **Compound** | **Deviance** | **P-value** |
| --- | --- | --- | --- |
| Nitrogen compounds | Propanaldoxime, 2-methyl, anti- | 3.471 | 0.001 |
|  | Benzyl nitrile | 10.970 | 0.018 |
|  | Butyl aldoxime, 2-methyl-, anti- | 17.683 | 0.003 |
|  | Butyl aldoxime, 2-methyl-, syn- | 22.644 | 0.001 |
|  | Butyl aldoxime, 3-methyl-, syn- | 19.461 | 0.001 |
|  | Indole | 2.477 | 0.001 |
| Benzenoids | Methyl benzoate | 7.522 | 0.007 |
|  | Benzyl alcohol | 8.152 | 0.018 |
| Monoterpenes | (E)-Geraniol | 2.326 | 0.001 |
|  | (Z)-*β-*Οcimene | 7.333 | 0.012 |
|  | (E)-*β-*Οcimene | 12.408 | 0.002 |
|  | D-Limonene | 2.396 | 0.001 |
|  | Geranyl acetate | 7.154 | 0.039 |
|  | *β*-Myrcene | 2.585 | 0.001 |

Table S3. Unique volatile compounds detected on the different floral parts of *C. spinosa.*

| **Floral part** | **Unique volatile compounds** |
| --- | --- |
| Calyx+Gynoecium | - |
| Corolla | 4-Penten-1-yl acetate^1^ |
|  | Caryophyllene^1^ |
|  | Hexyl acetate |
|  | Isoamyl alcohol |
|  | MO 119, 91, 134, 55, 43^1^ |
|  | SE 69, 41, 81, 93, 67^1^ |
|  | *α*-Farnesene |
| Nectar | 3-Heptanone |
|  | Benzaldehyde, 3-ethyl- |
| Stamens | Propanaldoxime, 2-methyl, syn- |
|  | MO 119, 91, 134, 55, 41 |
|  | MO 119, 91, 134, 55, 42^1^ |
|  | MO 119, 91, 134, 55, 44^1^ |

^1^Compound detected only in one sample of the respective group.

Table S5. Compounds that were present in three of the four floral parts sampled.

| **Floral part combination** | **Volatile compounds** |
| --- | --- |
| Calyx+Gynoecium/Corolla/Nectar | Isoamyl benzoate |
| Corolla/Nectar/Stamens | Isoamyl acetate |
|  | MO 121, 136, 93, 91, 79 |
| Calyx+Gynoecium/Corolla/Stamens | (E)-Geraniol |
|  | Geranyl acetate |
|  | Benzaldehyde |
|  | Benzyl pentanoate |
|  | BE 104, 43, 91, 105, 103 |
|  | Indole |
|  | Pentane, 1-nitro- |


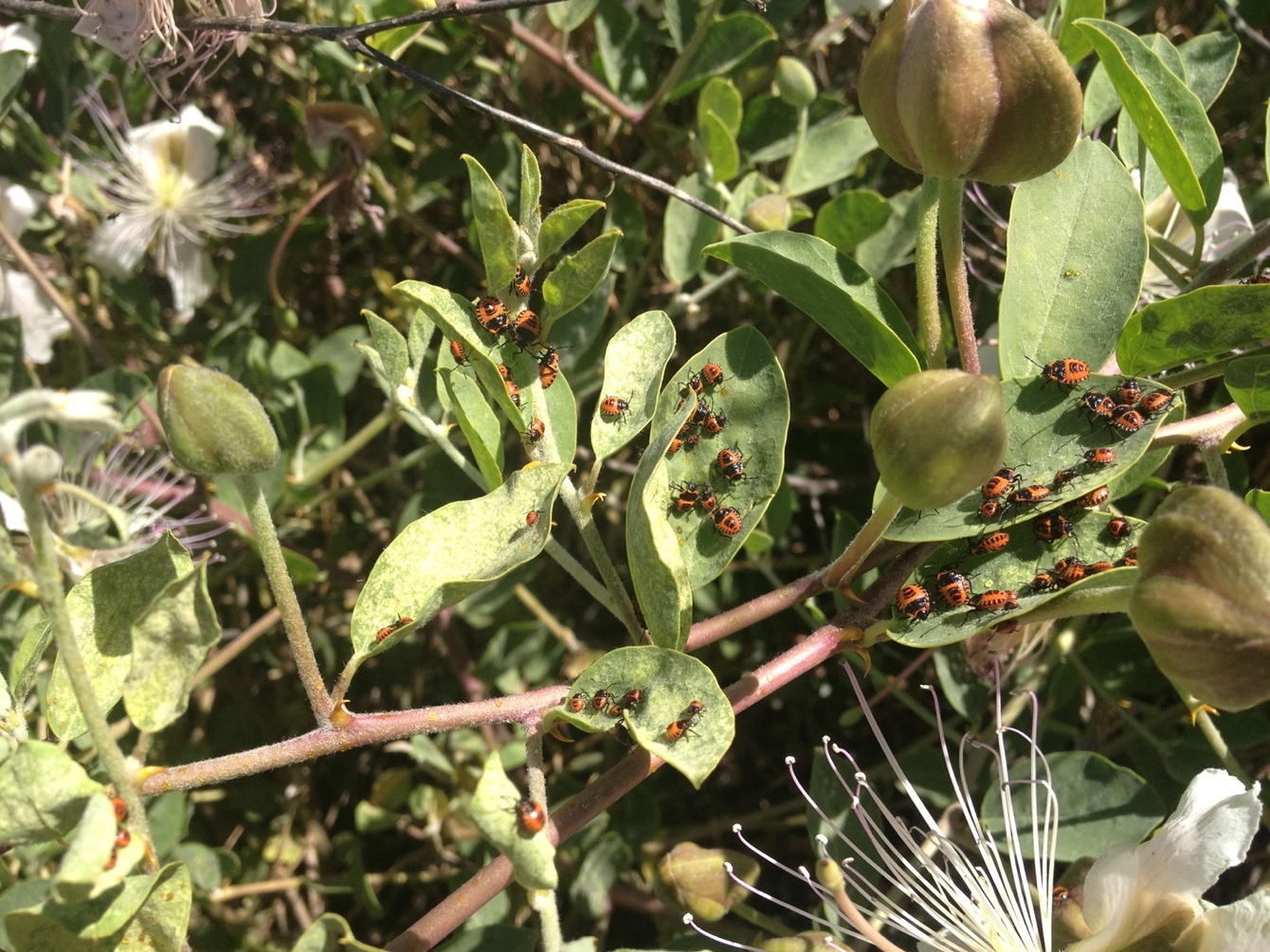


Figure S5. Adults and nymphs of *Eurydema* sp. (Pentatomidae) on *Capparis spinosa* on Lesvos Island, Greece. Photo: A. Kantsa.

Table S4. Floral visitors of *Capparis spinosa* recorded in June-July 1984-1985 at Daphni, Athens, Greece^[[1]](#footnote-1)^.

| **Order** | **Family** | **Species** |
| --- | --- | --- |
| Hymenoptera | Apidae | *Amegilla quadrifasciata* |
|  |  | *Xylocopa olivieri* |
|  |  | *Xylocopa violacea* |
|  |  | *Apis mellifera* |
|  | Halictidae | *Halictus resurgens* |
|  |  | *Halictus smaragdulus* |
|  |  | *Lasioglossum anellum* |
|  |  | *Lasioglossum limbelloides* |
|  |  | *Lasioglossum lucidulum* |
|  |  | *Lasioglossum malachurum* |
|  |  | *Lasioglossum politum politum* |
|  |  | *Lasioglossum pygmaeum patulum* |
|  | Megachilidae | *Anthidium florentinum* |
|  |  | *Rhodanthidium septemdentatum* |
| Coleoptera | Bruchidae | *Spermophagus sericeus* |
|  | Buprestidae | *Anthaxia* sp*.* |
|  |  | *Anthaxia umbellatarum* |
|  | Dasytidae | *Dasytes tristiculus* |

1. **Petanidou T.** **1991**. *Pollination ecology in a phryganic ecosystem,* PhD Thesis, Aristotle University of Thessaloniki, Thessaloniki. [↑](#footnote-ref-1)
